# Feasibility of dynamic structural equation modeling for capturing micro-level temporal dynamics in adolescent physical activity

**DOI:** 10.1186/s12889-026-26321-8

**Published:** 2026-01-17

**Authors:** Franziska Beck, Anne Kerstin Reimers, Ulrich Dettweiler

**Affiliations:** 1 Department of Sport Science and Sport, Friedrich-Alexander-Universität Erlangen-Nürnberg, Gebbertstraße 123B, Erlangen, 91052 Germany; 2 Norwegian Centre for Learning Environment and Behavioral Research in Education, University of Stavanger, Stavanger, 4036 Norway

**Keywords:** Feasibility study, Dynamic structural equation modeling, Physical activity, Adolescents, Temporal dynamics, Habitual behavior, Situational locus of control

## Abstract

**Background:**

Worldwide, physical activity (PA) levels among adolescents remain insufficient, highlighting the need for effective interventions. To improve these efforts, a nuanced understanding of habitual PA patterns and their fluctuations is essential. This feasibility study demonstrates the application of Dynamic Structural Equation Modeling (DSEM) to analyze micro-level temporal dynamics in adolescent PA behavior. Building on data from Beck et al. (2024) (*BMC Public Health*), one-week diary-based activity records from 15 adolescents (8 boys, 7 girls; mean age = 13.04 ± 1.28 years) were re-analyzed to examine: (1) the degree to which actual PA deviates from habitual PA, (2) the temporal dynamics of PA, and (3) the situational locus of control regulating these deviations.

**Methods:**

Participants first outlined their usual weekly schedules and then documented their actual behaviors for one week. This intensive longitudinal design yielded 1,785 hourly observations and descriptive data of habitual and actual PA was analyzed. A two-stage analytic approach was employed. First, given the small sample (*N =* 15), we used an idiographic approach with DSEM to analyze each adolescent’s longitudinal data, employing simple models to capture within-person dynamics and summarizing results descriptively by counting credible intervals excluding zero, stratified by gender. Second, situational locus of control underlying deviations from habitual PA was assessed using Pearson residuals.

**Results:**

N-of-1 DSEM analyses showed that both habitual plans and actual activity were relatively stable, with strong autoregressive patterns, evening reductions, and consistent positive alignment between habitual PA and action; cross-lagged and weather effects were minimal, highlighting stable idiographic dynamics.Self-controlled increases in PA occurred mainly toward the end of school days and early weekend mornings, whereas self-controlled decreases were most prominent in late afternoons.

**Conclusion:**

This feasibility study highlights the utility of DSEM for modeling fine-grained behavioral dynamics in small adolescent samples. Findings underscore stable yet context-sensitive PA patterns and demonstrate DSEM’s value as a methodological framework for future research on adolescent PA.

**Supplementary Information:**

The online version contains supplementary material available at 10.1186/s12889-026-26321-8.

## Background

### Benefits of physical activity and prevalence

Physical activity (PA) is a crucial predictor of both mental and physical health in adolescents [12, 40]. Consequently, numerous initiatives to promote PA have been implemented across various settings, including schools, family environments, and local communities. However, the effectiveness of these efforts has varied significantly in both the short and long term, and overall results have been somewhat disappointing [17, 35]. Additionally, the World Health Organization [58] stated that global efforts seem to miss the goal and further action is needed. This is also evidenced by the fact that adolescents in many regions worldwide do not meet the World Health Organization recommendations [6, 26, 58]. Specifically, with increasing age, the prevalence of guideline compliance in adolescents seems to decrease [7, 42, 57]. These findings highlight the need for a deeper understanding of the dynamics of PA patterns and the variability of habitual PA behavior in a holistic way to enhance the efficacy of promoting PA in adolescents [14, 41].

This feasibility study contributes to this goal by demonstrating how Dynamic Structural Equation Modeling (DSEM) can be used to analyze intensive longitudinal PA data at the micro-level. By re-analyzing data from Beck et al. [9], we aim to showcase the potential of DSEM for capturing temporal dynamics and situational influences in adolescent PA behavior. Here, ‘feasibility’ refers specifically to the analytic application of DSEM to intensive longitudinal PA data, rather than to a formal evaluation of multiple modeling strategies or implementation procedures.

### Variability and stability of weekly PA

Week-to-week variability in PA offers valuable insights into the stability of habitual behavior. The issue of stability of PA is particularly relevant from a public health perspective as epidemiological studies highlight that continuous and stable activity behavior reduces the risk of coronary heart disease and other health risks [29, 51]. However, it is assumed that variability of PA occurs in everybody’s daily life as it is unlikely for an individual to engage in the exact same amount of activity within a defined time period.

In this context, modeling intra-individual fluctuations becomes essential for understanding behavioral rhythms. In general, various factors could affect variability of PA levels [23]. Some of these factors are cyclical, that is, they repeat over time, such as daily (school, work, spare time), weekly (e.g., regular training in clubs), or seasonal patterns (e.g. higher PA during warmer seasons, cf. [19]) and these factors are likely to contribute to the overall variability over time. Others are not cyclical, for example, weather, diseases, life events and transitions, active holidays, or age [23, 25, 41].

Findings on the stability of habitual PA behavior are mixed. Some research in adults has documented strong weekly regularities, suggesting that activity patterns may become internalized and predictable over time [41]. On the other hand, a study in children has reported only moderate to low week-to-week reliability in habitual movement and postural behaviors under free-living conditions [30], indicating that developmental stages and contextual variability may limit stability in younger populations.

Together, this underscores the importance of studying intra-individual variability in PA behavior, particularly in adolescents, whose routines may be shaped by both structured and unstructured contexts. Understanding how PA fluctuates across time and situations can inform the design of interventions that align with adolescents’ lived rhythms and behavioral tendencies.

### Hourly assessment of PA and underlying dynamics

While weekly variability provides insights into broader patterns, analyzing PA dynamics on an hourly basis is crucial for understanding the structure of PA behavior within a day. To the best of our knowledge, there are only a few studies that focus on this level of detailed temporal analysis. Research using high-resolution accelerometer data remains limited, and early studies [36, 37] primarily described hourly differences in activity levels—for example, elevated PA during school recess or after-school hours. Although informative, these studies are now dated and did not examine associations between consecutive time points, leaving the dynamic structure of PA largely unexplored.

While more existing studies have assessed hourly PA (mostly using accelerometry), they typically analyzed PA levels in children and adolescents within different segments of the day, such as before school, during school/physical education, recess, and after school [31, 46, 47, 53]. For example, one of these studies showed that school hours and early evening tend to be the most active periods, whereas sedentary behavior peaks in the late evening [16]. However, these studies did not focus on the different hours within these segments and only indicated a mean value of PA within these time periods and consequently the specific associations between consecutive time points (hours) could not be established. To deepen the understanding of the dynamics of PA, it is important to assess those associations in within analyses and to evaluate the effects of such different variables as age, gender or weather. For instance, a study focusing on youth PA patterns during different segments throughout the day showed that activity levels in youth vary during the day, and in terms of gender and season [43].

### Situational locus of control

Besides the mentioned cyclical and non-cyclical factors that have an influence of PA patterns [23, 25, 41], another important factor for influencing variability and deviations in habitual PA behavior is the situational locus of control. This concept describes the extent to which individuals believe life events are determined by their own actions (internal locus) or external factors such as chance or other people (external locus) [24, 33]. Unlike the general locus of control, which is considered a stable personality trait, the situational locus of control can vary depending on the specific context or situation. Individuals may perceive more agency over their activity levels when they attribute control to their own actions (internal locus) in a given situation, while in other situations, they may attribute deviations to uncontrollable factors such as chance or external influences (external locus) [24, 33]. Recent evidence supports the notion that individuals with a more internal health-related locus of control exhibit higher self-control and are more likely to engage in and maintain PA [13]. Moreover, among adolescents, internal health locus of control was shown to influence PA not only directly but also indirectly via self-efficacy and perceived social support [59]. These findings suggest that perceptions of control play an important role in PA engagement. However, no existing studies have examined how situational, momentary control perceptions relate to short-term deviations from habitual PA patterns or micro-temporal PA dynamics. Incorporating situational locus of control into intensive longitudinal models such as DSEM could therefore help to explain when and why adolescents maintain or deviate from their habitual PA levels. Understanding this dynamic mechanism provides a theoretically grounded and empirically promising pathway for identifying situational factors that regulate fluctuations in daily PA behavior.

### Use of Dynamic Structural Equation Modeling (DSEM) in prior research on behavioral dynamics

Beyond segment-based or hour-level analyses, a small but growing body of research has begun applying DSEM to study the temporal dynamics of health-related behaviors. These studies demonstrate the potential of DSEM to capture within-person fluctuations and bidirectional associations in intensive longitudinal data. For instance, Armstrong et al. [1] used DSEM to examine day-to-day changes in sleep, sedentary behavior, and moderate-to-vigorous PA on school and non-school days in children and adolescents. Similarly, Bedree et al. [10] employed DSEM to analyze bidirectional links between daily pain and PA in emerging adults, highlighting how contextual factors shape moment-to-moment behavioral patterns.

In summary, there are several notable research gaps regarding PA in children and adolescents. Despite numerous interventions across schools, families, and communities, the effectiveness of these efforts remains inconsistent, and a deeper understanding of the dynamics of PA patterns is needed to enhance their efficacy. Although research on week-to-week variability in PA exists, it primarily focuses on adults or general observations, leaving a gap in understanding specific influencing factors in children and adolescents. Additionally, most studies assess PA in segmented time periods (e.g., school hours, after school) rather than providing detailed hourly insights or examining how activity levels in one hour influence subsequent hours. Further, analyses of within-person dynamics over time controlled for possible time sensitive and time-invariant variables are lacking. Another unexplored area is the role of situational locus of control in PA behavior, which could provide valuable insights into how personal agency and external factors drive deviations from habitual patterns. Lastly, while there exist studies that illustrate the methodological value of DSEM for modeling dynamic processes, none have investigated hourly PA fluctuations in adolescents under consideration of soziodemographic and weather data. This underscores the potential of applying DSEM to better understand micro-level PA dynamics in youth.

Addressing these gaps could significantly advance the understanding and promotion of PA in younger populations.

As illustrated, there are notable research gaps concerning the dynamics of PA levels in adolescents—particularly regarding intra-day fluctuations and situational variability. While some studies have addressed week-to-week patterns, few have examined PA behavior on an hourly basis using intensive longitudinal designs. We can thus formulate the following research questions: (1) How are adolescents’ habitual PA levels reflected in their actual PA, (2) what are the micro-dynamics of habitual and actual PA, (3) what is the situational locus of control that regulates possible deviations from habitual PA behaviors?

## Methods

### Study design

**Fig. 1:**
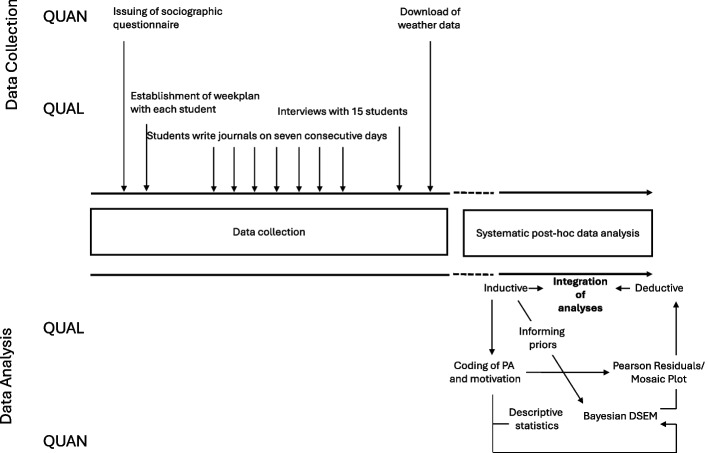
Data collection and data analyzes of the present study

This study does not aim to produce generalizable findings, but rather to demonstrate the feasibility and utility of DSEM for analyzing micro-level PA data in small samples. It is conceived as a cross-sectional study; however, it included a longitudinal within-subjects component, with follow-up conducted hourly over one week. We applied a mixed approach, drawing on qualitative and quantitative data, and integrating them in comprehensive statistical models (see Fig. 1). The study was approved by the local Ethics Committee of the Friedrich-Alexander-Universität Erlangen-Nürnberg (Ref. No. 23–31-S) and was in accordance with the 1964 Declaration of Helsinki (version of 2013). All participants and their legal guardians provided written informed consent for study participation.

### Participants

Study participants were adolescents aged 11–15 years living in Germany. Recruitment took place via personal contacts, various youth institutions as well as sports clubs and other leisure time instances in the region of Erlangen (Bavaria). We used the approach of theoretical sampling methods [38], focusing on a diverse sample with respect to socioeconomic status, migration status, sex/gender and environmental conditions (e.g., urban and rural living locations). More specifically, we tried to find a similar number of boys and girls for each category (sex/gender, age, migrant background, area of residence and type of school). Our final sample consisted of 15 adolescents (see Table 1 for more information). Adolescents were excluded from the study if they had a disability or did not own a smartphone. All participants received a 10 Euro incentive.

**Table 1:** Sample description

	Overall	Boys	Girls
Adolescents			
N (%)	15 (100)	8 (53)	7 (47)
Age (mean SD)	13.04 (1.28)	12.99 (1.46)	13.10 (1.17)
11	3	2 (66.7)	1 (33.3)
12	4	2 (50.0)	2 (50.0)
13	5	2 (40.0)	3 (60.0)
14	2	1 (50.0)	1 (50.0)
15	1	1 (100)	0
School type			
Middle School (Haupt/Mittelschule)	1 (7)	1(12.5)	0
Secondary School (Realschule)	8 (53.3)	4 (50.0)	4 (57.1)
High School (Gymnasium)	6 (40)	3 (37.5)	3 (42.9)
Urbanisation			
City > 100.000 inhabitants	5 (33)	2 (25.0)	3 (42.8)
Medium sized town 20.000–100.000 inhabitants	4 (27)	1 (12.5)	3 (42.8)
Rural area/village	6 (40)	5 (62.5)	1 (14.4)
Migration Background			
None	13 (87)	6 (75.0)	7 (100)
One custodian	1 (7)	1 (12.5)	0
Both custodian	1 (7)	1 (12.5)	0
Activity Level			
Diary MET Min per day (mean; standard deviation) Range (min – max)	661.0 (293.6) 227.3–1175.4	796.4 (304.9) 389.1–1175.4	506.3 (183.4) 227.3–724.1
Custodians			
Educational Level of custodian 1			
(qualified) middle school degree	4 (26.7)	2(25.0)	2 (28.6)
High school degree	11 (73.3)	6 (65.0)	5 (62.5)
Educational Level of custodian 2			
Finished school without degree	1 (7.1)	1 (12.5)	0
Middle school degree	6 (42.9)	3 (37.5)	3(50.0)
General high school degree	7 (50.0)	4 (50.0)	3 (50.0)
Employment of custodian 1			
Part-time	9 (60.0)	3 (37.5)	6 (85.8)
Full-time	6 (40.0)	5 (62.5)	1 (14.2)
Employment of custodian 2			
Unemployed	1 (6.7)	1 (12.5)	0
Retiree	1 (6.7)	1 ()	0
Part-time	1 (6.7)	1 (12.5)	0
Full-time	11 (79.9)	5 (62.5)	6 (100)

### Data collection

Data collection took place in spring 2023 and consisted of three parts, (1) a questionnaire establishing the habitual PA, (2) a one-week online diary, (3) individual interviews, however, for the present study, only part one and two are included. After fulfilling a short paper–pencil-survey on sociodemographic data in the first phase, participants provided a detailed weekly schedule including all physical activities within one habitual/typical week. Therefore, a plain document where they could fulfill the template with their activities was sent to the participants per email (see Additional File 1). Examples were given. However, if adolescents needed assistance completing the habitual schedule, they could contact our research team or seek help from their parents. The second part was represented by an activity diary over one week, which was conducted after the first part. For easy handling we chose a smartphone-based diary app of the company ClueTec GmbH (https://www.cluetec.de/solutions/mquest/mquest-diary-software/). To use the diary, adolescents received a QR-code that made an installation of the programmed diary available. Then they could track their activities easily on their smartphone. To minimize recall bias, adolescents were instructed to record their activities ideally immediately after they occurred. If this wasn't possible, they were advised to do so as soon as possible afterward. All participant data was de-identified to ensure anonymity.

### Measures

#### Sociodemographic data

Adolescents were asked about their age, gender and school they were currently visiting. The parent’s questionnaire contained questions on residential area, highest educational level, as well as current occupation and migration status.

#### Weekly activity schedule (habitual PA or INTHAB)

Participants provided information about their habitual physical activities within one week. They were supposed to deliver information about the time of the activity (clock time), the duration (derived from the time of the activities), type (naming the kind of activity), and intensity (indicating level of perceived intensity from sleeping (0) to maximum intensity (4)). For better understanding they received a template containing two examples (see Additional File 1).

#### Smartphone based activity diary (actual PA or INTACT)

Participants were asked to track their activities with the smartphone-based diary app. For every PA they were doing, they were asked to track these activities in the app and indicate time of the activity (clock time), the type (naming the kind of activity), duration (in minutes), and intensity (indicating level of perceived intensity from sleeping (0) to maximum intensity (4)) (see Additional File 2).

#### Temperature and precipitation

We retrieved weather data, i.e., hourly temperature and precipitation averages, individually for each child from the German Weather Service [21]. Temperature was given as degrees Celsius [°C] and precipitation was indicated with mm per hour. Data were collected from the weather station that was nearest to the adolescents’ home.

#### Situational locus of control

Within the diary, we gained a lot of situational information like accompanionship, decision maker for the activity as well as performed activities before and after.

### Data preparation and coding

#### Age

The age of the adolescents was coded as months for the analysis.

#### PA levels

Adolescents rated their habitual behavior within the weekly schedule in five levels as sleeping, awake but sedentary (SB), light PA (LPA), moderate PA (MPA) or vigorous PA (VPA). Within the activity diary, they only recorded their active physical behavior (e.g., 5:30–07:15 pm, football training, very exhaustive) and their sleeping hours verbally, which were then coded by the researchers using again a 5-point Likert scale from zero for “sleeping” to four for VPA time slots without any activities were coded with 1. When different PA-levels occurred during one hour, e.g., the football training ending at 07:15 pm and the remaining 45 min in this time interval was blank, we still coded this interval with the higher PA level (in this example, “4”).

#### Situational locus of control

Based on the situational information within the diaries, the deviation from the habitual behavior as well as the locus of control were coded in six categories:

1. habitual/expected PA (no deviation) (e.g., habitual training session at Tₓ that was also performed during the diary week at Tₓ)
2. habitual/expected physical inactivity (no deviation) (e.g., a habitual school lesson at Tₓ that was included in the adolescent’s diary week at Tₓ)
3. self-controlled increase in PA (e.g., no habitual PA anticipated at Tₓ, yet the diary showed that the adolescent independently decided to engage in play in the garden at Tₓ),
4. self-controlled decrease in PA (e.g., habitual horse riding at Tₓ, yet the diary indicated reduced PA at Tₓ because the adolescent chose to shorten the session due to motivational factors)
5. externally controlled increase in PA (e.g., no habitual PA anticipated at Tₓ, yet the diary showed that the adolescent played with friends at their request Tₓ),
6. externally controlled decrease in PA (e.g., habitual football training at Tₓ occurred for a shorter duration as reported in the diary at Tₓ, because other teams used the field)

#### Time period

We clustered the hourly time intervals into three time periods, morning (including 6am-12 pm), afternoon (12 pm-6 pm), evening (6 pm-11 pm) and created a timestamp with running hours indexing the i = 119 observations for each individual. The hours from 11 to 6am were not included in the statistical analysis, as all of the adolescents indicated sleeping during this time period but were accounted for in the time-series analysis as described below.

#### Temperature and precipitation

Are conceived as numeric values but due to their different scales included in the models as z-standardized variables.

### Data analysis

Descriptive statistics were calculated for all study variables, with means (M) and standard deviation (SD) for continuous variables; hereby, we assumed a simplex structure of the PA-levels and treated the respective categories as equally distanced. For the statistical analysis of the quantitative data, we used a two-stage approach (see Fig. 1).

**Fig. 2:**
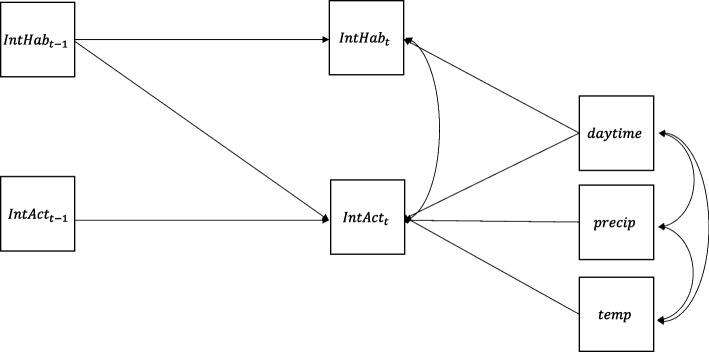
Simplified path diagram of the single-level Dynamic Structural Equation Model (DSEM) applied in this study. The model includes three time-varying predictors—daytime (categorical with three levels: morning, afternoon, evening), temperature (temp), and precipitation (precip)—as well as an autoregressive component for the outcome variables, habitual (IntHab) and actual (IntAct) intensity of physical activity. Rectangles denote observed variables in the autoregressive part and predictors. For clarity, error terms are omitted from the diagram; the simplified notation focuses on the dynamic relations among observed variables

For the inferential analyses, we initially specified multilevel Dynamic Structural Equation Models (DSEM) in Mplus (vs. 8.6, Muthen & Muthen), separately for each period of the day (morning, afternoon, evening) since we expected different dynamics in these day times due to school and spare time requirements. We then iteratively simplified the models by removing predictors that did not contribute substantively to the dynamic associations, following the recommendations of Asparouhov and Muthen [3]. Variables removed during this process included migration background, socioeconomic factors, and weekday. However, because the between-person sample size was very small (*N =* 15), estimating reliable multilevel parameters was not feasible. Consequently, we adopted an idiographic modeling approach and analyzed each adolescent’s intensive longitudinal data separately using DSEM. At this stage, between-level variables such as age and gender were, of course, excluded from the models. Time of day (morning, afternoon, evening) was included as a categorical predictor (see Fig. 2).

The individuals’ dynamic deviations were analyzed by estimating autoregressive relations using time-lagged (t–1) variables as predictors [27]. This approach allowed for analyzing the extent to which a preceding measurement occasion influenced subsequent measurement occasions at the concurrent time. To accommodate the long overnight gap, we modelled physical activity (PA) as a continuous autoregressive process (φ) whose carry‑over naturally decays with elapsed time (φ^Δt^; operationally, we coded hourly spacing and used Mplus TINTERVAL = 1 so that unobserved nighttime segments are treated as missing values estimated within the Bayesian DSEM framework, preserving continuity between the last evening and first morning observations [2]. This approach is consistent with the continuous-time interpretation of autoregressive processes, where longer intervals imply weaker lagged effects [54]. A recently proposed alternative is to estimate a separate overnight carry‑over (γ) from the last beep of day d–1 to the first beep of day d, explicitly testing whether night dynamics differ from daytime inertia; however, γ is identified only by day‑boundary pairs and, as shown by Berkhout, Schuurman, and Hamaker’s simulations, typically requires ≥ 100 days per individual for stable evidence, with “continues overnight” and “stops overnight” becoming empirically indistinguishable when Δt is long or φ is modest [11]. Given our idiographic design (*N =* 15) and limited days (D = 7), estimating γ would add uncertainty without improving inference, whereas continuity‑with‑decay via TINTERVAL is parsimonious and theoretically defensible for PA, and consistent with the discrete/continuous‑time equivalence for unequal intervals.

Model quality was determined using effective sample size (ESS), which estimated the amount by which autocorrelation in samples increases uncertainty relative to an independent sample [20]; and the potential scale reduction factor Rhat, which tested for non-stationarity with a simulation chain by comparing the distributions of each chain [15]. Bayesian estimation was conducted using two parallel chains with 15,000 iterations each and a 50% burn-in. Convergence was evaluated using standard criteria: For the final models all Rhat were ≤ 1.05, and all ESS exceeded 1,000, indicating satisfactory convergence.

Following Gigerenzer’s argument that slightly biased but frugal models can outperform general-purpose strategies when observations are limited, we summarized results descriptively by counting how often 95% credible intervals excluded zero across individuals, stratified by gender, without aggregating into misleading group-level estimates [22].

To evaluate the situational locus of control for deviations from the habitual PA behavior, we then calculated the Pearson residuals of the respective categories (A-F) for each hour. In contrast to the DSEM, we averaged here over the schooldays and the weekend, respectively, since we assumed different locus of control factors in those two weekly segments.

### Prior probabilities

The parameters of greatest substantive interest in this study were the autoregressive and cross-lagged slopes for the two PA variables, which were log-transformed prior to analysis. Setting priors on these transformed variables required careful consideration to ensure plausible ranges while avoiding undue constraint.

Although the within-person time series provided sufficient observations for estimation, these dynamic parameters can exhibit high variability and occasional extreme estimates under noninformative priors, particularly when predictors are correlated. To mitigate this risk while maintaining flexibility, we specified weakly informative Normal priors centered at zero with a variance of 0.25 (SD = 0.5). This choice reflects three considerations:

7. After log transformation, the plausible range of autoregressive and cross-lagged effects is narrower than on the raw scale. A prior variance of 0.25 allows slopes up to approximately ± 1.0 within two standard deviations, which is generous given theoretical expectations and empirical evidence for PA dynamics.
8. The prior is weak enough to permit meaningful positive or negative effects but discourages implausibly large values that could distort interpretation. This is particularly important for dynamic models, where extreme estimates can propagate instability across lags.
9. These slopes capture the core temporal processes of interest. Applying weakly informative priors here improves estimation robustness without imposing strong assumptions, while other parameters (e.g., intercepts, residual variances, and covariances) rely on Mplus defaults. For regression coefficients, defaults assume Normal distributions with infinite variance, and for variance parameters, Inverse-Wishart priors (conceptualized as inverse gamma distributions) are used, which are appropriate for strongly identified residuals.

Finally, to improve computational efficiency and maintain consistency, we vectorized priors for classes of parameters (e.g., autoregressive slopes) using a single specification applied across individuals. This approach simplifies code and ensures uniform prior assumptions for parameters serving similar roles.

## Results

### Data quality

Completing the habitual weekly schedule required approximately 30 min for the adolescents. Each activity recorded in the activity diary took adolescents between 2 to 5 min. The weekly schedules were comprehensive, without any time gaps. In the activity diary, only PA was recorded. If any entries were found to be missing in the diary, which were supposed to be included (e.g., active travel to school), participants were individually contacted to ascertain whether the activity had not occurred or had simply been forgotten.

### Sample characteristics

The mean age of the 15 adolescents (8 boys and 7 girls) was 13.04 (SD = 1.28). Approximately half of the participants (*N =* 6; 3 boys and 3 girls) attended a secondary school (Realschule), while the majority (*N =* 8; 4 boys and 4 girls) attended high school (Gymnasium). Only one boy from a middle school was included. A gender imbalance was observed in the residential distribution, with 5 boys and only 1 girl living in rural areas. Most of the adolescents' parents were born in Germany, except two participants having either a one-sided migration background (*N =* 1) or a two-sided migration background (*N =* 1). The adolescents recorded 661.0 (293.6) MET-minutes per day in their diaries, indicating an active sample (moderate physical activity = 3–6 MET-minutes, vigorous physical activity > 6 MET-minutes) [49]. Further information can be seen in Table 1.

### Descriptive data of PA levels and weather data

Overall, mean intensity level of habitual and actual behavior across the whole day was between sedentary behavior (awake) and light PA (INTHAB = 2.3 (0.9), INTACT = 2.23 (0.73)). For both, habitual and actual behavior, highest intensities were in the afternoon (INTHAB = 2.68 (0.84), INTACT = 2.43 (0.73)). Due to different time spans (End of April 2023 – Beginning of June 2023) as well as the daytime (6am – 23pm) of data collection, the temperature ranged overall from 1.5 to 28.2°C (mean 12.55 (4.03)) (see Table 2).

**Table 2:** Descriptive values for habitual and actual intensity levels as well as for temperature across the whole day as well as across morning, afternoon and evening time periods

	**Overall Mean (SD)**	**Morning Mean (SD)**	**Afternoon Mean (SD)**	**Evening Mean (SD)**
INTHAB	2.30 (0.90)	2.21 (0.79)	2.68 (0.84)	1.93 (0.92)
INTACT	2.23 (0.73)	2.22 (0.68)	2.43 (0.73)	2.01 (0.73)
DIFF	−0.06 (0.75)	0.01 (0.69)	−0.26 (0.8)	0.08 (0.73)
TEMP	12.55 (4.03)	11.73 (3.67)	14.58 (3.74)	11.1 (3.8)
PRECIP	0.06 (0.26)	0.11 (0.34)	0.04 (0.27)	0.02 (0.1)

^*^INTHAB habitual intensity, *INTACT* actual intensity, *DIFF* INTACT – INTHAB, *TEMP* Temperature, *PERCIP* Precipitation. INTHAB and INTACT range from 0–4 (0 = sleeping, 1 = awake but inactive, 2 = Light PA, 3 = Moderate PA, 4 = Vigorous PA); Morning: 6am-12pm, Afternoon: 12pm-6pm, Evening: 6pm-11pm

### Idiographic patterns in dynamic associations: the Dynamic Structural Equation Modell (DSEM)

The N-of-1 dynamic structural equation models revealed striking consistency in the direction of significant effects across individuals (see Table 3). Habitual activity intensity exhibited strong autoregressive persistence: INTHAB ON INTHAB_t-1_ was positive for 14 of 15 adolescents, indicating that once plans were set, they tended to carry forward hour-to-hour. Evening emerged as a systematic low point for habitual PA, with INTHAB ON EVENING negative in 11 cases, suggesting reduced intentions later in the day. Actual activity also showed inertia, with INTACT ON INTACT_t-1_ positive in 9 cases—more frequent among males (6 of 8) than females (3 of 7). Cross-lagged effects from habitual to subsequent action **(**INTACT ON INTHAB_t-1_**)** were positive wherever significant (6 cases), appearing more often among females (4 of 7) than males (2 of 8). Evening reductions extended to actual activity: INTACT ON EVENING was negative in all 6 significant cases (2 females, 4 males). Weather effects were minimal: Temperature never predicted actual intensity; precipitation predicted actual intensity in one case (ID 8, positive). Across all participants, afternoons were warmer and evenings cooler relative to mornings; precipitation and temperature were negatively associated for IDs 7 and 10 only. Finally, the contemporaneous association between the habitual PA and action (INTHAB WITH INTACT) was positive in 13 adolescents, underscoring a robust same-hour alignment between intentions and behavior. Importantly, the direction of these effects was consistent across all individuals, highlighting stable idiographic dynamics rather than random variability (see Additional File 3 for graphical displays of the data and the parameter estimates of the 15 individual models).

**Table 3:** N-of-1 dynamic structural equation models

Parameter	Girls (*N =* 7): # IDs (+/–)	Boys (*N =* 8): # IDs (+/–)	Total significant
INTHAB on INTHAB&1 (AR of habitual)	**7** (7/0)	**7** (7/0)	14
INTHAB on afternoon	1 (1/0)	3 (3/0)	4
INTHAB on evening	**6** (0/6)	**5** (0/5)	11
INTACT on INTACT&1 (AR of actual)	3 (3/0)	**6** (6/0)	9
INTACT on INTHAB&1 (cross-lag)	**4** (4/0)	2 (2/0)	6
INTACT on evening	2 (0/2)	**4** (0/4)	6
INTACT on PERC	0 (0/0)	1 (1/0)	1
INTHAB with INTACT (concurrent association)	**6** (+)	**7** (+)	13

^*^Each cell shows how many individuals of that gender had a significant effect for the parameter; “ + ”/“–” indicate the direction of the significant estimate. Parameters are included only if they were significant for at least one ID. Significance is reached when the posterior 95% credible interval does not include zero

### Chi-squared and pearson residual analysis

**Fig. 3:**
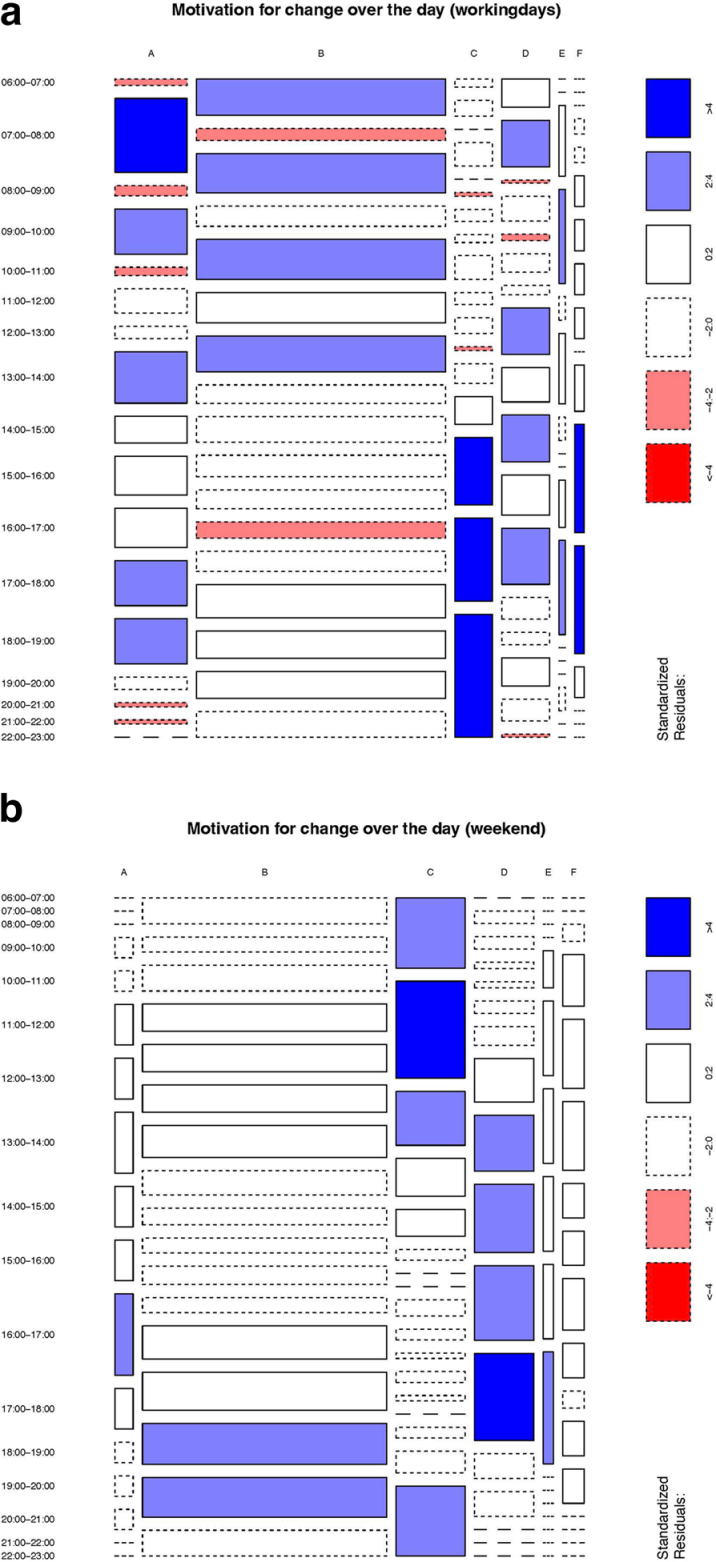
**a **Mosaic Plot for situational locus of control that regulated potential deviation for school days. A) habitual/expected PA (no deviation), B) habitual/expected physical inactivity (no deviation), C) self-controlled increase in PA, D) self-controlled decrease in PA, E) externally controlled increase in PA, F) externally controlled decrease in PA. **b** Mosaic Plot for situational locus of control that regulated potential deviation for weekend days. A) habitual/expected PA (no deviation), B) habitual/expected physical inactivity (no deviation), C) self-controlled increase in PA, D) self-controlled decrease in PA, E) externally controlled increase in PA, F) externally controlled decrease in PA

The analysis of the categorical data about the children’s situational locus of control that regulates potential deviations clearly showed that self-controlled increase of PA was prominent towards the end of the days during school and early in the morning on weekends, whereas self-controlled decrease of PA with respect to habitual activity was the dominant behavior in the later afternoon, both on school days and on weekends. One can also see that the categories that represent self-controlled behavior were statistically overrepresented on the weekends, while there was a clear pattern of change between externally and self-controlled behavior on the school days: whenever school and family obligations theoretically allowed self-controlled behavior, the self-controlled categories were dominant. Hereby, habitual inactivity was the dominant behavior during school hours, while habitual PA was more dominant for the school ways (to and from) and early in the evening, when most adolescents engaged in organized sports (see Fig. 3a and b).

## Discussion

This study did not aim to compare alternative models or assess implementation feasibility as in a clinical or intervention context. Rather, by re-analyzing the dataset from Beck et al. [9], we aimed to explore the potential of DSEM for capturing micro-level temporal dynamics and situational variability in PA behavior. Our data indicated that most adolescents (13 out of 15) stuck to their habitual behavior with only slight deviations in the afternoon. In the descriptive data we see overall a minimal difference (mean −0.06) between daily habitual and actual intensity of activity behavior. Similarly, a study focusing on variability in weekly PA of adults by assessing steps per day over one year found a relatively stable weekly rhythm of steps per day and thus confirms our findings within the present study [41]. Further, a study with children also identified a low week to week variability [30], that is confirmed by our study. This is in line with the assumption, that PA behavior follows a cyclic rhythm within a week and this weekly pattern repeats every week [23, 41].

Nevertheless, we found a difference between habitual and actual PA levels in the afternoon, even if this difference is still small (mean difference = −0.26 (0.088). Our data indicated that the afternoon time period is the one with the highest PA levels (see descriptive data Table 2), allowing for greater deviations compared to morning and evening where less habitual activity is accumulated. Even if the highest PA levels are reached in the afternoon time period, it seems to be the most unstructured time period [32]. This allows adolescents to deviate from/decrease their PA levels and these deviations were predominantly self-determined in our study which is due to the fact that most compulsory activities such as school and organized PA tend to take place in the morning or evening (see Mosaic Plot, Fig. 3a).

Even if we found just one significant difference between habitual and actual PA levels in the afternoon, the analysis of situational locus of control delivers additional complementing information about the regulation of deviations from the habitual PA behavior: Overall, the differences in regulation between weekdays and weekends become apparent, with self-controlled behavior being statistically overrepresented on weekends, while weekdays show a clear pattern of alternation between externally controlled and self-controlled behavior. In more detail, on weekdays the morning time period is dominated by expected/habitual (in)activity due to sedentary behavior during school time [8]. Further, in the evening (6 pm-11 pm) adolescents either participate in organized sports or stay at home (data based on diary), resulting in less opportunities to vary their own PA behavior [36]. This pattern aligns with the finding that the expected/habitual activity behavior tends to be the dominant behavior in the evening time period on school days. On weekend, the mosaic plot indicated predominantly self-controlled behavior. Weekends generally exhibit greater variability in behavior, reflecting the increased freedom to choose activities [56]. While research indicates that PA levels often decline during weekends due to fewer opportunities for organized or externally directed activities [4, 48], the less structured nature of this time can also facilitate spontaneous or leisure-driven PA, depending on individual preferences and available environmental opportunities.

Focusing on habitual and actual behavior separately, the autoregression indicated significant associations between PA at timepoint t and timepoint t-1 in both time series, habitual and actual PA. This suggests a PA pattern where past activity levels influence future activity levels, which accounts for a large consistency in adolescents' PA behaviors. For habitual PA with significant and positive autoregression effects among 14 of 15 adolescents, this indicates a high degree of hour-to-hour stability in habitual activity tendencies, suggesting that once a certain habitual activation level is present, it is likely to persist across consecutive hours. In the context of actual PA, autocorrelation was significant in 60% of the adolescents. This indicates both a lack of spontaneity in the adolescents’ PA behaviors, but it also means that high (or low) PA at one time point positively predicts high (or low) activity at the next. Similar results can be seen in the descriptive data in the study of Mota et al. [36], with constant peaks in each time period (9am-12 pm, 12 pm-3 pm, 3 pm-6 pm, 6 pm-9 pm). Interestingly, this association was more often prominent among boys (6 of 8) than females (3 of 7), pointing to stronger persistence in actual PA behavior in boys [55].

Despite the overall hour-to-hour stability within our data, we found that habitual as well as actual PA behavior tends to drop in the evening compared to the morning. This pattern could be explained by adolescents’ daily schedules. In the morning, adolescents may engage in active travel to school and participate in PA during school recess and physical education classes. In contrast, in the evening (18:00–22:00) they are predominantly at home, where PA levels tend to be lower and sedentary behaviour higher [34, 37, 39].

The observed positive cross-lagged association between habitual activity intensity and actual activity intensity in the subsequent hour likely reflects habitual behavioral continuity rather than deliberate self-regulation. At this short time scale, PA appears to be driven primarily by automated, context-dependent routines, resulting in stability of activity intensity across adjacent time points [18, 52].

Lastly, in one case, precipitation predicted actual intensity while temperature had not reached significance for any individual and was left out in the final model. Other studies stated that the weather is a strong predictor of PA levels and thus children’s PA levels fluctuate across the year [5, 50]*.* The difference between our study and existing studies could be explained by the data collection period: In the present study the whole data collection took place within two months in spring 2023 with small differences in temperature within and between the subjects (mean temperature 12.55 °C (4.03)) and did not focus on different seasons like winter and summer where greater temperature differences are expected. The mentioned existing studies assessed PA levels over one year and thus included all seasons [5, 50].

### Implications

With our study we wanted to gain deeper insights how to efficiently increase the effectiveness of PA promotion in adolescents by analyzing activity patterns. For developing interventions, the hourly analysis could give important information: Our data indicated that the afternoon is the least stable period during the day compared to morning and evening where obligations like school classes or organized sports activities take place. However, the afternoon is the most unstructured part of the day [36] and could thus be benefit from more structure in form of PA interventions (e.g., after-school programs) [28, 36]. As seen in the morning during school time as well as in the early evening, adolescents stuck to their behavior as this is kind of mandatory (school) and organized PA is habitual and kind of hedonistic activity [9]. Following, we assume that a habitual PA program in the afternoon can help to internalize this behavior and thus to increase overall PA levels.

Furthermore, PA levels in the morning of the participants of our studies were low, especially in girls. Having this in mind, we suggest improving opportunities to be active during school recess and implement active elements into classes [45].

### Strengths and limitations

The present study has several strengths. First of all, we investigated habitual and actual behavior, which allows new insights into the internalization and patterns of PA in adolescents. Thus, focusing on time-sensitive differences and autoregression analysis within days using cutting-edge statistical models provides novel insights. Even though we included only 15 participants, we generated intensive longitudinal data of 17 h per day across one week (119 pairs of actual and habitual PA behavior for each of the adolescents which make up 1785 data points). Further, we used an activity diary to get more information about each PA behavior that allows a deeper understanding of activity behavior. Importantly, this study serves as a methodological proof-of-concept for applying DSEM to intensive longitudinal PA data in small samples. The results demonstrate that meaningful insights into behavioral dynamics can be gained even with limited N, provided the data are rich and temporally structured. Moreover, we suggest that DSEM can be applied to analyze PA data irrespective of the mode of data collection. In our case, the data were obtained through self-reported diary entries, but the same modeling approach is equally applicable to objective measures such as accelerometer data. With sufficiently large samples, the DSEM framework can be extended into a multilevel model that preserves intraindividual dynamics while simultaneously capturing between-individual variability and could later test whether the overnight carry‑over (γ) truly differs from daytime inertia (φ).

This flexibility makes DSEM a promising tool for future PA research across diverse methodological designs, enabling researchers to model intra-individual variability and contextual influences with high temporal resolution.

However, there are some limitations that need to be discussed. Firstly, the sample size (*N =* 15) is relatively small which doesn’t justify DSEM analysis on the between level. Further due to the data collection method using a smartphone-based app, adolescents without smartphones were unable to participate. Regarding measurement methods, the habitual weekly schedule was assessed only once during a single week, which may have resulted in inflated reports of PA due to social desirability bias. Moreover, this approach lacked the capacity to fully validate habitual behavior, as it did not incorporate longitudinal data or objective measures to confirm the consistency of reported routines. Additionally, the subjective and retrospective nature of assessing activities within both the weekly schedule and the diary introduces a significant risk of recall bias, potentially affecting the accuracy and reliability of the data. Another limitation is that we only analyzed the intensities of the activities on a 5-level scale from sleeping to vigorous activity. Lastly, we coded and analyzed the activities on an hourly basis and not the exact time spans. In this context, we also divided the day into three time periods (morning, afternoon and evening) instead of a structure like before school, schooltime, afterschool etc. Nevertheless, we believe to have made a significant step to analyze the structural dynamics of adolescents’ activity patterns. Future studies should find solutions to mitigate the limitations of this research.

## Conclusion

As a feasibility study, this work demonstrates the utility of DSEM for modeling adolescent PA behavior at a micro-level. The findings suggest that even small-N designs can yield valuable insights into behavioral rhythms and situational influences, paving the way for future studies with larger samples and extended timeframes. Employing a mixed-methods approach, we identified a stable activity pattern among all participants, with afternoon periods showing more variability compared to mornings and evenings, possibly due to the unstructured nature in this time period. Further, our data indicated in the habitual and actual PA levels a significant autoregression between PA at timepoint t-1 and t, suggesting a PA pattern where past activity levels influence future activity levels, which accounts for a large consistency in adolescents' PA behaviors. In terms of intervention implications, we propose incorporating programs during the afternoon period and focusing on internalization to ensure sustained behavioral impact comparable to organized sports participation.

## Supplementary Information

Supplementary Material 1.

Supplementary Material 2. 

Supplementary Material 3.

## Data Availability

The datasets used and/or analysed during the current study are available from the corresponding author on reasonable request.
