# Supplementary material for: Feasibility of dynamic structural equation modeling for capturing micro-level temporal dynamics in adolescent physical activity
Source: BMC Public Health. 2026 Jan 17;26:345. doi: 10.1186/s12889-026-26321-8 (PMC12849192; doi:10.1186/s12889-026-26321-8)
Supplement: Supplementary file 2 — Supplementary Material 2. [file 12889_2026_26321_MOESM2_ESM.pdf]

Additional File 2: Questions from the smartphone-based activity diary

---

Dear study participant,

Thank you for taking part in the study.
The next step is for you to record all your activities over one week. Please make a **new entry for each physical activity** (walking, sports, cycling, etc.).
If the space is not enough, simply copy and paste the questions for each activity.

Sedentary activities do not need to be recorded!

Date Activity 1: ____________

1. When did you go to bed yesterday? _________
2. When did you get up today? ___________
3. What time did your activity take place? ____________
4. How long did the activity last? ______ min
5. How intense was the activity?

    ☐ light

    ☐ moderate

    ☐ maximum

6. Location of the activity: _________________________
7. Weather (multiple answers possible):

    ☐ Sun

    ☐ Clouds

    ☐ Rain

    ☐ Thunderstorm

    ☐ Wind

    ☐ Storm

    ☐ Fog

8. Temperature (multiple answers possible):

- ☐ very hot
- ☐ hot
- ☐ warm
- ☐ cold
- ☐ very cold

9. Who was present? (multiple answers possible):

- ☐ nobody
- ☐ friends (number: ______)
- ☐ coach/trainer
- ☐ teacher
- ☐ parents
- ☐ siblings
- ☐ other: _______

10. If others were present, what were they doing? ___________________

11. Who decided that you would do this activity?

- ☐ myself
- ☐ others: _______________
- ☐ fixed appointment during the week

12. How much effort did you put in?

- ☐ none
- ☐ a little
- ☐ some
- ☐ a lot

13. What was particularly exhausting? _______________________________________

14. Would you have liked to put in more effort?

☐ yes → how could this have been possible?
____

☐ no

15. Did you have fun?

☐ yes

☐ no

16. What was especially good? ___________________________________
17. What was not so good? ________________________________
18. Can you remember what you were thinking about during the activity?

☐ yes, namely ___________________________________

☐ no

19. What did you do before the activity? ________________________________
20. What did you do after the activity? _______________________________
21. Mood before the activity:

☐ good

☐ okay

☐ bad

Note: ____________________

22. Mood after the activity:

☐ good

☐ okay

☐ bad

Note: ____________
