# Supplementary material for: Feasibility of dynamic structural equation modeling for capturing micro-level temporal dynamics in adolescent physical activity
Source: BMC Public Health. 2026 Jan 17;26:345. doi: 10.1186/s12889-026-26321-8 (PMC12849192; doi:10.1186/s12889-026-26321-8)
Supplement: Supplementary file 3 — Supplementary Material 3. [file 12889_2026_26321_MOESM3_ESM.pdf]

Additional File 3:

Feasibility of Dynamic Structural Equation Modeling for Capturing Micro-Level Temporal Dynamics in Adolescent Physical Activity

Franziska Beck[1*], Anne Kerstin Reimers[1] & Ulrich Dettweiler[2]

[1] Department of Sport Science and Sport, Friedrich-Alexander-Universität Erlangen-Nürnberg, Gebbertstraße 123b, 91052 Erlangen, Germany

[2] Norwegian Centre for Learning Environment and Behavioral Research in Education, University of Stavanger, 4036 Stavanger, Norway

*Corresponding Author: Franziska Beck, franzi.beck@fau.de

Supplement

1. Descriptive Plots

1.1. The Difference between the Intensity of Habitual (Planned) and Actual Physical Activity

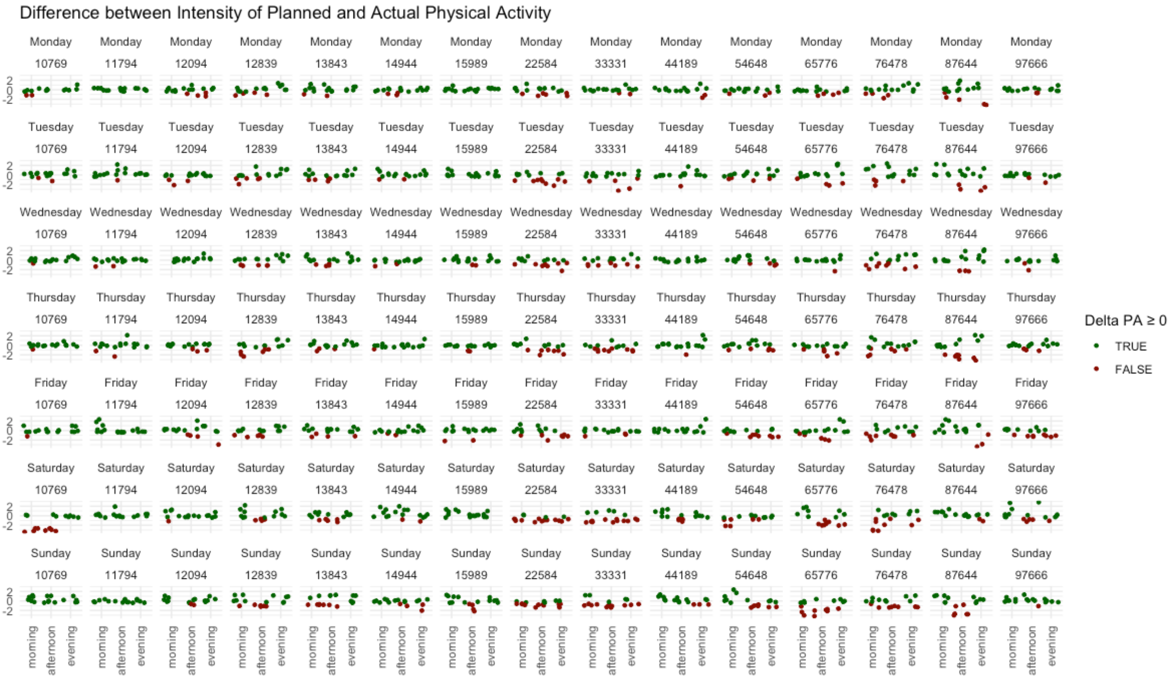

2. Weather Data

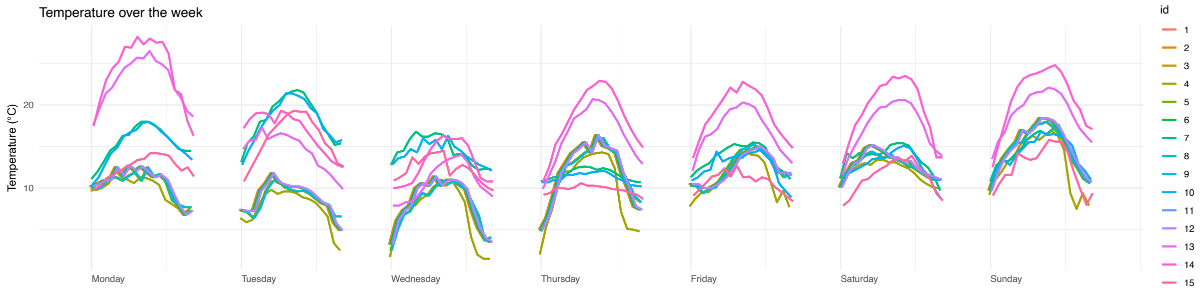

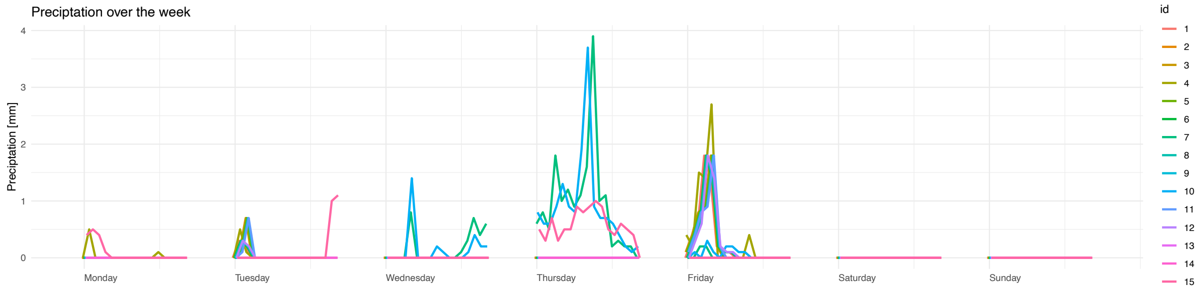

For ID's 13-15, no precipitation was registered during the week.

3. Parameter Estimates of the 15 Individual DSEMs (INTPLAN = INTHAB)

3.1. ID 1: Female

STDYX Standardization

	Estimate	Posterior S.D.	One-Tailed P-Value	95% C.I. Lower 2.5%	95% C.I. Upper 2.5%	Significance
INTPLAN ON						
INTPLAN&1	0.644	0.079	0.000	0.486	0.796	*
AFTERNOON	0.105	0.081	0.090	-0.046	0.271	
EVENING	-0.193	0.076	0.010	-0.334	-0.033	*
INTACT ON						
INTACT&1	0.326	0.098	0.001	0.131	0.513	*
INTPLAN&1	0.200	0.119	0.046	-0.030	0.437	
AFTERNOON	0.057	0.098	0.279	-0.136	0.249	
EVENING	-0.154	0.094	0.056	-0.334	0.034	
TEMP	0.092	0.072	0.096	-0.045	0.239	
PERC	0.004	0.064	0.473	-0.125	0.127	
INTPLAN WITH						
INTACT	0.628	0.059	0.000	0.498	0.727	*
PERC WITH						
TEMP	-0.084	0.096	0.196	-0.269	0.104	
AFTERNOO WITH						
TEMP	0.458	0.076	0.000	0.294	0.593	*
PERC	-0.126	0.093	0.089	-0.304	0.060	
EVENING WITH						
TEMP	-0.303	0.087	0.000	-0.469	-0.129	*
PERC	-0.127	0.094	0.096	-0.306	0.064	
AFTERNOON	-0.477	0.074	0.000	-0.614	-0.322	*

Means						
TEMP	-0.007	0.091	0.464	-0.188	0.168	
PERC	-0.003	0.092	0.489	-0.178	0.180	
AFTERNOON	0.698	0.103	0.000	0.494	0.898	*
EVENING	0.628	0.101	0.000	0.434	0.837	*
Intercepts						
INTPLAN	0.569	0.190	0.000	0.241	0.984	*
INTACT	1.044	0.251	0.000	0.580	1.560	*
Variances						
TEMP	1.000	0.000	0.000	1.000	1.000	
PERC	1.000	0.000	0.000	1.000	1.000	
AFTERNOON	1.000	0.000	0.000	1.000	1.000	
EVENING	1.000	0.000	0.000	1.000	1.000	
Residual Variances						
INTPLAN	0.506	0.099	0.000	0.303	0.692	*
INTACT	0.675	0.092	0.000	0.477	0.832	*

MCMC EFFECTIVE SAMPLE SIZE (ESS) IN ASCENDING ORDER
LOWEST 10 PARAMETERS

PARAMETER	ESS
9	3378
1	4970
14	5396
2	6197
8	8460
17	9291
15	9574
13	9728
5	9856
7	10038

3.2. ID 2: Female

STDYX Standardization

	Estimate	Posterior S.D.	One-Tailed P-Value	95% C.I. Lower 2.5%	Upper 2.5%	Significance
INTPLAN ON						
INTPLAN&1	0.478	0.082	0.000	0.317	0.638	*
AFTERNOON	0.045	0.087	0.301	-0.121	0.220	
EVENING	-0.388	0.080	0.000	-0.534	-0.219	*
INTACT ON						
INTACT&1	0.251	0.094	0.004	0.062	0.427	*
INTPLAN&1	0.217	0.101	0.013	0.020	0.421	*
AFTERNOON	0.049	0.092	0.288	-0.131	0.232	
EVENING	-0.400	0.082	0.000	-0.554	-0.231	*
TEMP	0.046	0.064	0.231	-0.077	0.175	
PERC	-0.003	0.058	0.478	-0.119	0.108	
INTPLAN WITH						
INTACT	0.665	0.055	0.000	0.544	0.755	*
PERC WITH						
TEMP	-0.100	0.095	0.151	-0.283	0.087	
AFTERNOO WITH						
TEMP	0.458	0.076	0.000	0.294	0.593	*
PERC	-0.148	0.093	0.054	-0.324	0.038	
EVENING WITH						
TEMP	-0.303	0.088	0.000	-0.468	-0.130	*
PERC	-0.132	0.094	0.085	-0.311	0.057	
AFTERNOON	-0.477	0.074	0.000	-0.613	-0.321	*
Means						
TEMP	-0.008	0.091	0.464	-0.186	0.170	
PERC	-0.003	0.092	0.485	-0.178	0.178	
AFTERNOON	0.698	0.103	0.000	0.497	0.900	*

EVENING	0.632	0.101	0.000	0.439	0.840	*
**Intercepts**						
INTPLAN	1.082	0.220	0.000	0.654	1.522	*
INTACT	1.387	0.248	0.000	0.915	1.889	*
**Variances**						
TEMP	1.000	0.000	0.000	1.000	1.000	
PERC	1.000	0.000	0.000	1.000	1.000	
AFTERNOON	1.000	0.000	0.000	1.000	1.000	
EVENING	1.000	0.000	0.000	1.000	1.000	
**Residual Variances**						
INTPLAN	0.585	0.075	0.000	0.434	0.728	*
INTACT	0.584	0.069	0.000	0.447	0.715	*

MCMC EFFECTIVE SAMPLE SIZE (ESS) IN ASCENDING ORDER

LOWEST 10 PARAMETERS

PARAMETER	ESS
9	4059
1	4960
2	6387
14	8592
6	9440
7	9478
5	9531
17	10094
15	10471
18	10531

3.3. ID 3: Female

STDYX Standardization

	Estimate	Posterior S.D.	One-Tailed P-Value	95% C.I. Lower 2.5%	Upper 2.5%	Significance
**INTPLAN ON**						
INTPLAN&1	0.466	0.086	0.000	0.300	0.634	*
AFTERNOON	0.126	0.091	0.077	-0.050	0.306	
EVENING	-0.311	0.083	0.000	-0.465	-0.139	*
**INTACT ON**						
INTACT&1	0.027	0.094	0.391	-0.159	0.210	
INTPLAN&1	0.323	0.112	0.002	0.104	0.549	*
AFTERNOON	0.033	0.097	0.361	-0.156	0.223	
EVENING	-0.414	0.085	0.000	-0.570	-0.233	*
TEMP	0.080	0.060	0.084	-0.036	0.200	
PERC	0.023	0.054	0.338	-0.086	0.127	
**INTPLAN WITH**						
INTACT	0.740	0.045	0.000	0.641	0.815	*
**PERC WITH**						
TEMP	-0.100	0.095	0.151	-0.283	0.089	
**AFTERNOO WITH**						
TEMP	0.459	0.076	0.000	0.297	0.593	*
PERC	-0.148	0.093	0.055	-0.327	0.037	
**EVENING WITH**						
TEMP	-0.303	0.087	0.000	-0.469	-0.129	*
PERC	-0.132	0.094	0.085	-0.310	0.057	
AFTERNOON	-0.478	0.074	0.000	-0.615	-0.321	*
**Means**						
TEMP	-0.010	0.091	0.456	-0.189	0.168	
PERC	-0.002	0.092	0.489	-0.178	0.180	
AFTERNOON	0.694	0.103	0.000	0.493	0.895	*
EVENING	0.633	0.101	0.000	0.440	0.841	*
**Intercepts**						
INTPLAN	1.066	0.235	0.000	0.615	1.543	*
INTACT	1.348	0.239	0.000	0.879	1.831	*

```
 Variances
    TEMP               1.000       0.000       0.000       1.000       1.000
    PERC               1.000       0.000       0.000       1.000       1.000
    AFTERNOON          1.000       0.000       0.000       1.000       1.000
    EVENING            1.000       0.000       0.000       1.000       1.000

 Residual Variances
    INTPLAN            0.615       0.075       0.000       0.464       0.756      *
    INTACT             0.638       0.071       0.000       0.493       0.774      *

     MCMC EFFECTIVE SAMPLE SIZE (ESS) IN ASCENDING ORDER
        LOWEST 10 PARAMETERS
        PARAMETER    ESS
             9      3314
             1      4145
             2      4539
            14      5267
             5      8795
             7      8880
            17      9524
             6      9701
            12      9813
            18     10095
```

3.4. ID 4: Male

```
STDYX Standardization

                                Posterior  One-Tailed         95% C.I.
                    Estimate       S.D.      P-Value   Lower 2.5%  Upper 2.5%  Significance
 INTPLAN    ON
    INTPLAN&1          0.621       0.088       0.000       0.447       0.789      *
    AFTERNOON          0.125       0.085       0.062      -0.031       0.297
    EVENING           -0.174       0.081       0.026      -0.320       0.001

 INTACT     ON
    INTACT&1           0.163       0.093       0.041      -0.022       0.339
    INTPLAN&1          0.325       0.123       0.003       0.083       0.564      *
    AFTERNOON         -0.036       0.108       0.374      -0.242       0.181
    EVENING           -0.050       0.108       0.320      -0.258       0.167
    TEMP               0.076       0.087       0.190      -0.095       0.241
    PERC              -0.055       0.074       0.226      -0.203       0.084

 INTPLAN  WITH
    INTACT             0.512       0.071       0.000       0.357       0.631      *

 PERC     WITH
    TEMP              -0.027       0.096       0.395      -0.216       0.160

 AFTERNOO WITH
    TEMP               0.520       0.071       0.000       0.367       0.644      *
    PERC              -0.130       0.093       0.082      -0.308       0.056

 EVENING  WITH
    TEMP              -0.439       0.078       0.000      -0.581      -0.280      *
    PERC              -0.130       0.094       0.089      -0.309       0.058
    AFTERNOON         -0.478       0.074       0.000      -0.613      -0.320      *

 Means
    TEMP              -0.010       0.091       0.453      -0.189       0.166
    PERC              -0.002       0.092       0.491      -0.179       0.182
    AFTERNOON          0.696       0.103       0.000       0.495       0.898      *
    EVENING            0.630       0.101       0.000       0.435       0.834      *

 Intercepts
    INTPLAN            0.621       0.206       0.000       0.267       1.071      *
    INTACT             2.097       0.369       0.000       1.393       2.847      *

 Variances
    TEMP               1.000       0.000       0.000       1.000       1.000
    PERC               1.000       0.000       0.000       1.000       1.000
    AFTERNOON          1.000       0.000       0.000       1.000       1.000
    EVENING            1.000       0.000       0.000       1.000       1.000

 Residual Variances
```

INTPLAN	0.536	0.109	0.000	0.310	0.735	*
INTACT	0.760	0.096	0.000	0.541	0.908	*

MCMC EFFECTIVE SAMPLE SIZE (ESS) IN ASCENDING ORDER
LOWEST 10 PARAMETERS

PARAMETER	ESS
9	3383
1	4767
14	5484
8	6775
13	9158
5	9568
7	9705
12	10719
2	10815
3	10873

3.5. ID 5: Female

STDYX Standardization

	Estimate	Posterior S.D.	One-Tailed P-Value	95% C.I. Lower 2.5%	Upper 2.5%	Significance
INTPLAN ON						
INTPLAN&1	0.497	0.071	0.000	0.361	0.636	*
AFTERNOON	0.152	0.079	0.025	-0.001	0.310	
EVENING	-0.445	0.073	0.000	-0.579	-0.293	*
INTACT ON						
INTACT&1	0.105	0.093	0.130	-0.074	0.282	
INTPLAN&1	0.129	0.098	0.094	-0.067	0.316	
AFTERNOON	-0.006	0.122	0.480	-0.242	0.234	
EVENING	-0.124	0.109	0.128	-0.336	0.093	
TEMP	-0.074	0.102	0.234	-0.270	0.124	
PERC	-0.081	0.095	0.190	-0.271	0.099	
INTPLAN WITH						
INTACT	0.064	0.095	0.257	-0.130	0.241	
PERC WITH						
TEMP	-0.113	0.095	0.121	-0.294	0.076	
AFTERNOO WITH						
TEMP	0.463	0.076	0.000	0.300	0.597	*
PERC	-0.147	0.093	0.055	-0.327	0.040	
EVENING WITH						
TEMP	-0.241	0.091	0.004	-0.417	-0.062	*
PERC	-0.130	0.094	0.089	-0.310	0.057	
AFTERNOON	-0.480	0.074	0.000	-0.614	-0.325	*
Means						
TEMP	-0.014	0.091	0.441	-0.193	0.169	
PERC	-0.003	0.092	0.486	-0.179	0.179	
AFTERNOON	0.687	0.103	0.000	0.485	0.889	*
EVENING	0.644	0.101	0.000	0.451	0.846	*
Intercepts						
INTPLAN	1.026	0.189	0.000	0.648	1.383	*
INTACT	4.382	0.626	0.000	3.131	5.555	*
Variances						
TEMP	1.000	0.000	0.000	1.000	1.000	
PERC	1.000	0.000	0.000	1.000	1.000	
AFTERNOON	1.000	0.000	0.000	1.000	1.000	
EVENING	1.000	0.000	0.000	1.000	1.000	
Residual Variances						
INTPLAN	0.452	0.059	0.000	0.342	0.572	*
INTACT	0.902	0.049	0.000	0.783	0.970	*

MCMC EFFECTIVE SAMPLE SIZE (ESS) IN ASCENDING ORDER
LOWEST 10 PARAMETERS

PARAMETER	ESS
9	6074
1	7051
14	8964
5	9086
6	9818
7	10467
25	10925
28	11051
27	11184
12	11296

3.6. ID 6: Female

STDYX Standardization

	Estimate	Posterior S.D.	One-Tailed P-Value	95% C.I. Lower 2.5%	95% C.I. Upper 2.5%	Significance
INTPLAN ON						
INTPLAN&1	0.550	0.085	0.000	0.384	0.713	*
AFTERNOON	0.216	0.090	0.007	0.043	0.392	*
EVENING	-0.169	0.083	0.029	-0.321	0.005	
INTACT ON						
INTACT&1	0.080	0.104	0.217	-0.127	0.278	
INTPLAN&1	0.298	0.125	0.007	0.052	0.539	*
AFTERNOON	0.094	0.112	0.198	-0.125	0.312	
EVENING	-0.181	0.101	0.040	-0.375	0.024	
TEMP	0.017	0.079	0.416	-0.138	0.172	
PERC	0.003	0.072	0.485	-0.142	0.140	
INTPLAN WITH						
INTACT	0.549	0.069	0.000	0.396	0.666	*
PERC WITH						
TEMP	-0.083	0.096	0.198	-0.268	0.104	
AFTERNOO WITH						
TEMP	0.459	0.076	0.000	0.294	0.594	*
PERC	-0.126	0.093	0.088	-0.304	0.060	
EVENING WITH						
TEMP	-0.303	0.088	0.001	-0.469	-0.128	*
PERC	-0.127	0.094	0.095	-0.305	0.063	
AFTERNOON	-0.479	0.074	0.000	-0.614	-0.321	*
Means						
TEMP	-0.013	0.091	0.443	-0.190	0.166	
PERC	-0.002	0.092	0.491	-0.177	0.181	
AFTERNOON	0.689	0.103	0.000	0.485	0.890	*
EVENING	0.633	0.101	0.000	0.439	0.840	*
Intercepts						
INTPLAN	0.900	0.249	0.000	0.442	1.414	*
INTACT	1.565	0.336	0.000	0.924	2.247	*
Variances						
TEMP	1.000	0.000	0.000	1.000	1.000	
PERC	1.000	0.000	0.000	1.000	1.000	
AFTERNOON	1.000	0.000	0.000	1.000	1.000	
EVENING	1.000	0.000	0.000	1.000	1.000	
Residual Variances						
INTPLAN	0.573	0.087	0.000	0.395	0.732	*
INTACT	0.778	0.084	0.000	0.584	0.910	*

MCMC EFFECTIVE SAMPLE SIZE (ESS) IN ASCENDING ORDER

LOWEST 10 PARAMETERS

PARAMETER	ESS
9	3974
1	4839
14	4911
2	5103

```
           7      9042
           5      9148
           8      9338
          12      9618
          17      9632
          13      9904
```

3.7. ID 7: Female

```
STDYX Standardization

                                Posterior  One-Tailed         95% C.I.
                   Estimate       S.D.      P-Value   Lower 2.5%  Upper 2.5%  Significance
 INTPLAN    ON
    INTPLAN&1          0.598       0.080      0.000       0.444       0.754      *
    AFTERNOON          0.087       0.086      0.150      -0.073       0.262
    EVENING           -0.252       0.079      0.001      -0.398      -0.086      *

 INTACT     ON
    INTACT&1           0.226       0.116      0.030      -0.008       0.444
    INTPLAN&1          0.403       0.117      0.000       0.171       0.631      *
    AFTERNOON          0.051       0.097      0.300      -0.135       0.248
    EVENING           -0.172       0.088      0.030      -0.337       0.008
    TEMP               0.050       0.080      0.251      -0.095       0.220
    PERC               0.000       0.064      0.499      -0.128       0.124

 INTPLAN  WITH
    INTACT             0.600       0.064      0.000       0.459       0.705      *

 PERC     WITH
    TEMP              -0.300       0.087      0.001      -0.461      -0.123      *

 AFTERNOO WITH
    TEMP               0.479       0.075      0.000       0.318       0.609      *
    PERC               0.098       0.094      0.153      -0.090       0.276

 EVENING  WITH
    TEMP              -0.287       0.088      0.001      -0.454      -0.113      *
    PERC              -0.108       0.094      0.131      -0.288       0.078
    AFTERNOON         -0.479       0.075      0.000      -0.614      -0.321      *

 Means
    TEMP              -0.017       0.092      0.425      -0.194       0.163
    PERC              -0.003       0.092      0.483      -0.181       0.176
    AFTERNOON          0.685       0.104      0.000       0.481       0.890      *
    EVENING            0.647       0.101      0.000       0.455       0.852      *

 Intercepts
    INTPLAN            0.769       0.213      0.000       0.380       1.216      *
    INTACT             0.623       0.239      0.000       0.200       1.144      *

 Variances
    TEMP               1.000       0.000      0.000       1.000       1.000
    PERC               1.000       0.000      0.000       1.000       1.000
    AFTERNOON          1.000       0.000      0.000       1.000       1.000
    EVENING            1.000       0.000      0.000       1.000       1.000

 Residual Variances
    INTPLAN            0.534       0.088      0.000       0.352       0.700      *
    INTACT             0.570       0.104      0.000       0.354       0.762      *

    MCMC EFFECTIVE SAMPLE SIZE (ESS) IN ASCENDING ORDER
        LOWEST 10 PARAMETERS
        PARAMETER    ESS
             2      2845
             9      2886
            14      4252
             1      4269
            13      5159
            15      5272
            12      6969
            10      7431
            18      7952
             8      7984
```

3.8. ID 8: Male

STDYX Standardization

	Estimate	Posterior S.D.	One-Tailed P-Value	95% C.I. Lower 2.5%	95% C.I. Upper 2.5%	Significance
INTPLAN ON						
INTPLAN&1	0.492	0.082	0.000	0.332	0.652	*
AFTERNOON	0.313	0.093	0.000	0.131	0.497	*
EVENING	-0.032	0.088	0.359	-0.200	0.150	
INTACT ON						
INTACT&1	0.445	0.080	0.000	0.282	0.586	*
INTPLAN&1	0.116	0.089	0.099	-0.062	0.291	
AFTERNOON	0.126	0.110	0.119	-0.084	0.352	
EVENING	-0.104	0.097	0.150	-0.291	0.090	
TEMP	-0.062	0.088	0.245	-0.236	0.111	
PERC	0.178	0.081	0.015	0.018	0.336	*
INTPLAN WITH						
INTACT	0.173	0.094	0.041	-0.022	0.346	
PERC WITH						
TEMP	-0.084	0.096	0.196	-0.269	0.106	
AFTERNOO WITH						
TEMP	0.458	0.076	0.000	0.294	0.593	*
PERC	-0.126	0.093	0.083	-0.306	0.061	
EVENING WITH						
TEMP	-0.303	0.087	0.001	-0.469	-0.128	*
PERC	-0.126	0.094	0.097	-0.305	0.063	
AFTERNOON	-0.476	0.074	0.000	-0.612	-0.319	*
Means						
TEMP	-0.007	0.091	0.469	-0.185	0.174	
PERC	-0.005	0.092	0.479	-0.183	0.178	
AFTERNOON	0.699	0.103	0.000	0.499	0.900	*
EVENING	0.627	0.102	0.000	0.432	0.832	*
Intercepts						
INTPLAN	0.654	0.193	0.000	0.292	1.046	*
INTACT	1.336	0.308	0.000	0.761	1.975	*
Variances						
TEMP	1.000	0.000	0.000	1.000	1.000	
PERC	1.000	0.000	0.000	1.000	1.000	
AFTERNOON	1.000	0.000	0.000	1.000	1.000	
EVENING	1.000	0.000	0.000	1.000	1.000	
Residual Variances						
INTPLAN	0.635	0.075	0.000	0.475	0.772	*
INTACT	0.660	0.074	0.000	0.504	0.794	*

MCMC EFFECTIVE SAMPLE SIZE (ESS) IN ASCENDING ORDER

LOWEST 10 PARAMETERS

PARAMETER	ESS
14	7257
9	7786
1	9009
2	9203
5	9479
12	9871
13	10542
18	10996
7	11316
3	11359

3.9. ID 9: Male

```
STDYX Standardization

                               Posterior  One-Tailed         95% C.I.
                    Estimate       S.D.      P-Value   Lower 2.5%  Upper 2.5%  Significance
 INTPLAN    ON
    INTPLAN&1          0.445       0.086      0.000       0.279       0.613      *
    AFTERNOON          0.201       0.087      0.010       0.035       0.368      *
    EVENING           -0.371       0.077      0.000      -0.514      -0.211      *

 INTACT     ON
    INTACT&1           0.361       0.084      0.000       0.191       0.517      *
    INTPLAN&1         -0.059       0.101      0.278      -0.245       0.148
    AFTERNOON          0.215       0.108      0.027      -0.003       0.423
    EVENING           -0.164       0.097      0.045      -0.352       0.028
    TEMP               0.033       0.090      0.350      -0.141       0.209
    PERC              -0.016       0.081      0.417      -0.178       0.137

 INTPLAN  WITH
    INTACT             0.328       0.086      0.001       0.144       0.479      *

 PERC     WITH
    TEMP              -0.113       0.095      0.119      -0.295       0.075

 AFTERNOO WITH
    TEMP               0.463       0.076      0.000       0.301       0.596      *
    PERC              -0.147       0.093      0.054      -0.326       0.038

 EVENING  WITH
    TEMP              -0.242       0.091      0.005      -0.418      -0.062      *
    PERC              -0.131       0.094      0.088      -0.311       0.058
    AFTERNOON         -0.480       0.074      0.000      -0.615      -0.324      *

 Means
    TEMP              -0.013       0.092      0.440      -0.193       0.166
    PERC              -0.003       0.092      0.488      -0.178       0.181
    AFTERNOON          0.687       0.103      0.000       0.483       0.888      *
    EVENING            0.640       0.101      0.000       0.447       0.845      *

 Intercepts
    INTPLAN            1.205       0.245      0.000       0.717       1.678      *
    INTACT             2.371       0.388      0.000       1.619       3.124      *

 Variances
    TEMP               1.000       0.000      0.000       1.000       1.000
    PERC               1.000       0.000      0.000       1.000       1.000
    AFTERNOON          1.000       0.000      0.000       1.000       1.000
    EVENING            1.000       0.000      0.000       1.000       1.000

 Residual Variances
    INTPLAN            0.535       0.070      0.000       0.398       0.671      *
    INTACT             0.726       0.066      0.000       0.586       0.842      *

    MCMC EFFECTIVE SAMPLE SIZE (ESS) IN ASCENDING ORDER
        LOWEST 10 PARAMETERS
        PARAMETER    ESS
            9      4519
            1      5249
           14      6092
            5      8800
            7      9232
            2      9380
            6     10098
           12     10907
           25     11056
           18     11303
```

3.10. ID 10: Male

```
STDYX Standardization

                               Posterior  One-Tailed         95% C.I.
                    Estimate       S.D.      P-Value   Lower 2.5%  Upper 2.5%  Significance
```

```
 INTPLAN    ON
    INTPLAN&1          0.532       0.074       0.000       0.388       0.678      *
    AFTERNOON         -0.074       0.081       0.185      -0.222       0.097
    EVENING           -0.461       0.080       0.000      -0.608      -0.295      *

 INTACT     ON
    INTACT&1           0.423       0.087       0.000       0.245       0.589      *
    INTPLAN&1          0.294       0.083       0.000       0.129       0.457      *
    AFTERNOON         -0.104       0.090       0.121      -0.284       0.076
    EVENING           -0.232       0.084       0.004      -0.396      -0.070      *
    TEMP               0.069       0.086       0.194      -0.087       0.248
    PERC               0.005       0.073       0.471      -0.136       0.149

 INTPLAN  WITH
    INTACT             0.326       0.087       0.000       0.138       0.480      *

 PERC     WITH
    TEMP              -0.270       0.089       0.002      -0.435      -0.091      *

 AFTERNOO WITH
    TEMP               0.454       0.077       0.000       0.289       0.591      *
    PERC               0.114       0.093       0.118      -0.072       0.291

 EVENING  WITH
    TEMP              -0.249       0.090       0.003      -0.422      -0.070      *
    PERC              -0.131       0.093       0.087      -0.311       0.057
    AFTERNOON         -0.479       0.075       0.000      -0.613      -0.321      *

 Means
    TEMP              -0.008       0.092       0.459      -0.187       0.173
    PERC              -0.003       0.092       0.483      -0.180       0.177
    AFTERNOON          0.696       0.103       0.000       0.496       0.901      *
    EVENING            0.639       0.100       0.000       0.448       0.843      *

 Intercepts
    INTPLAN            1.080       0.211       0.000       0.668       1.489      *
    INTACT             0.861       0.259       0.000       0.396       1.405      *

 Variances
    TEMP               1.000       0.000       0.000       1.000       1.000
    PERC               1.000       0.000       0.000       1.000       1.000
    AFTERNOON          1.000       0.000       0.000       1.000       1.000
    EVENING            1.000       0.000       0.000       1.000       1.000

 Residual Variances
    INTPLAN            0.515       0.070       0.000       0.380       0.654      *
    INTACT             0.520       0.081       0.000       0.362       0.679      *


    MCMC EFFECTIVE SAMPLE SIZE (ESS) IN ASCENDING ORDER
        LOWEST 10 PARAMETERS
        PARAMETER    ESS
             9      5167
             1      5963
             2      6382
            15      8643
            14      8679
             5     10256
            10     10389
             6     10472
            18     10544
             7     10819
```

3.11. ID 11: Female

```
STDYX Standardization

                                Posterior  One-Tailed         95% C.I.
                    Estimate       S.D.      P-Value   Lower 2.5%  Upper 2.5%  Significance
 INTPLAN    ON
    INTPLAN&1          0.663       0.072       0.000       0.523       0.802      *
    AFTERNOON          0.025       0.082       0.386      -0.128       0.194
    EVENING           -0.183       0.076       0.012      -0.327      -0.025      *

 INTACT     ON
```

```
   INTACT&1           0.475       0.074       0.000       0.328       0.614      *
   INTPLAN&1         -0.082       0.097       0.204      -0.255       0.128
   AFTERNOON          0.187       0.105       0.041      -0.020       0.391
   EVENING            0.068       0.100       0.250      -0.131       0.263
   TEMP              -0.078       0.070       0.129      -0.215       0.059
   PERC              -0.016       0.061       0.388      -0.140       0.100

 INTPLAN  WITH
   INTACT             0.712       0.050       0.000       0.602       0.794      *

 PERC     WITH
   TEMP              -0.099       0.096       0.151      -0.284       0.089

 AFTERNOO WITH
   TEMP               0.459       0.076       0.000       0.297       0.593      *
   PERC              -0.148       0.093       0.056      -0.326       0.040

 EVENING  WITH
   TEMP              -0.304       0.087       0.000      -0.469      -0.130      *
   PERC              -0.130       0.094       0.086      -0.310       0.057
   AFTERNOON         -0.478       0.074       0.000      -0.613      -0.322      *

 Means
   TEMP              -0.009       0.091       0.461      -0.188       0.171
   PERC              -0.003       0.092       0.484      -0.177       0.179
   AFTERNOON          0.697       0.103       0.000       0.493       0.902      *
   EVENING            0.632       0.101       0.000       0.436       0.837      *

 Intercepts
   INTPLAN            0.627       0.191       0.000       0.288       1.028      *
   INTACT             1.557       0.315       0.000       0.962       2.206      *

 Variances
   TEMP               1.000       0.000       0.000       1.000       1.000
   PERC               1.000       0.000       0.000       1.000       1.000
   AFTERNOON          1.000       0.000       0.000       1.000       1.000
   EVENING            1.000       0.000       0.000       1.000       1.000

 Residual Variances
   INTPLAN            0.510       0.090       0.000       0.323       0.673      *
   INTACT             0.755       0.062       0.000       0.618       0.859      *

   MCMC EFFECTIVE SAMPLE SIZE (ESS) IN ASCENDING ORDER
       LOWEST 10 PARAMETERS
       PARAMETER    ESS
           9      3343
          14      3442
           1      5005
           2      6354
          17      8147
          18      8516
           7      8634
          16      9286
           5      9869
          12     10337
```

3.12. ID 12: Male

```
STDYX Standardization

                                Posterior  One-Tailed         95% C.I.
                    Estimate       S.D.      P-Value   Lower 2.5%  Upper 2.5%  Significance
 INTPLAN    ON
   INTPLAN&1          0.652       0.089       0.000       0.468       0.819      *
   AFTERNOON          0.062       0.097       0.254      -0.104       0.267
   EVENING           -0.171       0.082       0.029      -0.316       0.007

 INTACT     ON
   INTACT&1           0.360       0.089       0.000       0.180       0.526      *
   INTPLAN&1          0.130       0.121       0.144      -0.101       0.371
   AFTERNOON         -0.032       0.116       0.386      -0.256       0.203
   EVENING           -0.150       0.105       0.086      -0.349       0.063
   TEMP              -0.016       0.079       0.420      -0.169       0.141
   PERC               0.025       0.072       0.358      -0.115       0.165
```

```
 INTPLAN  WITH
    INTACT            0.547       0.069       0.000       0.396       0.663      *

 PERC     WITH
    TEMP             -0.084       0.096       0.196      -0.269       0.105

 AFTERNOO WITH
    TEMP              0.459       0.076       0.000       0.295       0.595      *
    PERC             -0.126       0.094       0.090      -0.305       0.062

 EVENING  WITH
    TEMP             -0.304       0.087       0.000      -0.470      -0.127      *
    PERC             -0.126       0.094       0.097      -0.306       0.064
    AFTERNOON        -0.479       0.074       0.000      -0.615      -0.323      *

 Means
    TEMP             -0.012       0.091       0.449      -0.192       0.168
    PERC             -0.003       0.092       0.487      -0.178       0.180
    AFTERNOON         0.693       0.104       0.000       0.490       0.895      *
    EVENING           0.636       0.101       0.000       0.442       0.843      *

 Intercepts
    INTPLAN           0.672       0.209       0.000       0.308       1.126      *
    INTACT            2.093       0.392       0.000       1.360       2.898      *

 Variances
    TEMP              1.000       0.000       0.000       1.000       1.000
    PERC              1.000       0.000       0.000       1.000       1.000
    AFTERNOON         1.000       0.000       0.000       1.000       1.000
    EVENING           1.000       0.000       0.000       1.000       1.000

 Residual Variances
    INTPLAN           0.520       0.111       0.000       0.286       0.718      *
    INTACT            0.744       0.089       0.000       0.535       0.880      *


   MCMC EFFECTIVE SAMPLE SIZE (ESS) IN ASCENDING ORDER
        LOWEST 10 PARAMETERS
        PARAMETER    ESS
             9      3168
             1      5012
            14      5180
             7      5657
             8      5867
            13      8516
            12      8559
            17      9933
             5      9961
            18     10601
```

3.13. ID 13: Male (no percipitation)

```
STDYX Standardization

                                Posterior  One-Tailed         95% C.I.
                    Estimate       S.D.      P-Value   Lower 2.5%  Upper 2.5%  Significance
 INTPLAN    ON
    INTPLAN&1         0.483       0.096       0.000       0.291       0.667      *
    AFTERNOON         0.090       0.091       0.158      -0.083       0.273
    EVENING          -0.242       0.086       0.004      -0.403      -0.065      *

 INTACT     ON
    INTACT&1          0.249       0.093       0.004       0.064       0.433      *
    INTPLAN&1         0.195       0.110       0.036      -0.017       0.414
    AFTERNOON         0.009       0.098       0.466      -0.182       0.203
    EVENING          -0.245       0.091       0.005      -0.415      -0.056      *
    TEMP              0.059       0.081       0.231      -0.093       0.224

 INTPLAN  WITH
    INTACT            0.522       0.072       0.000       0.371       0.651      *

 AFTERNOO WITH
    TEMP              0.440       0.077       0.000       0.274       0.578      *
```

EVENING WITH						
TEMP	-0.234	0.088	0.004	-0.399	-0.053	*
AFTERNOON	-0.478	0.072	0.000	-0.606	-0.328	*
Means						
TEMP	-0.001	0.091	0.497	-0.184	0.173	
AFTERNOON	0.709	0.104	0.000	0.500	0.906	*
EVENING	0.640	0.101	0.000	0.446	0.839	*
Intercepts						
INTPLAN	1.101	0.281	0.000	0.587	1.688	*
INTACT	1.287	0.309	0.000	0.712	1.913	*
Variances						
TEMP	1.000	0.000	0.000	1.000	1.000	
AFTERNOON	1.000	0.000	0.000	1.000	1.000	
EVENING	1.000	0.000	0.000	1.000	1.000	
Residual Variances						
INTPLAN	0.664	0.093	0.000	0.466	0.832	*
INTACT	0.737	0.086	0.000	0.547	0.884	*

MCMC EFFECTIVE SAMPLE SIZE (ESS) IN ASCENDING ORDER

LOWEST 10 PARAMETERS

PARAMETER	ESS
8	3898
1	4487
2	4974
12	6108
7	9996
13	10107
5	10304
4	10465
16	10539
6	10656

3.14. ID 14: Male (no percipitation)

STDYX Standardization

	Estimate	Posterior S.D.	One-Tailed P-Value	95% C.I. Lower 2.5%	95% C.I. Upper 2.5%	Significance
INTPLAN ON						
INTPLAN&1	0.085	0.119	0.235	-0.150	0.321	
AFTERNOON	0.103	0.098	0.146	-0.091	0.293	
EVENING	-0.366	0.091	0.000	-0.532	-0.180	*
INTACT ON						
INTACT&1	-0.124	0.088	0.074	-0.303	0.046	
INTPLAN&1	0.219	0.139	0.058	-0.052	0.489	
AFTERNOON	-0.013	0.099	0.452	-0.209	0.185	
EVENING	-0.424	0.088	0.000	-0.584	-0.238	*
TEMP	0.060	0.048	0.098	-0.032	0.159	
INTPLAN WITH						
INTACT	0.890	0.020	0.000	0.844	0.923	*
AFTERNOO WITH						
TEMP	0.502	0.071	0.000	0.348	0.629	*
EVENING WITH						
TEMP	-0.288	0.086	0.000	-0.447	-0.113	*
AFTERNOON	-0.477	0.072	0.000	-0.609	-0.324	*
Means						
TEMP	0.002	0.091	0.492	-0.184	0.175	
AFTERNOON	0.715	0.104	0.000	0.506	0.912	*
EVENING	0.630	0.101	0.000	0.438	0.832	*
Intercepts						
INTPLAN	1.732	0.265	0.000	1.185	2.234	*
INTACT	1.806	0.264	0.000	1.259	2.285	*

```
 Variances
    TEMP               1.000       0.000       0.000       1.000       1.000
    AFTERNOON          1.000       0.000       0.000       1.000       1.000
    EVENING            1.000       0.000       0.000       1.000       1.000

 Residual Variances
    INTPLAN            0.791       0.065       0.000       0.651       0.906      *
    INTACT             0.769       0.066       0.000       0.628       0.889      *

MCMC EFFECTIVE SAMPLE SIZE (ESS) IN ASCENDING ORDER
        LOWEST 10 PARAMETERS
        PARAMETER    ESS
             8      1563
             1      1921
             2      1985
            12      2179
             5      7748
            15      8138
            14      8265
            16      8266
             6      8376
            10      8563
```

3.15. ID 15: Male (no precipitation)

```
STDYX Standardization

                                Posterior  One-Tailed         95% C.I.
                    Estimate       S.D.      P-Value   Lower 2.5%  Upper 2.5%  Significance
 INTPLAN    ON
    INTPLAN&1          0.556       0.075       0.000       0.403       0.699      *
    AFTERNOON          0.200       0.085       0.008       0.038       0.370      *
    EVENING           -0.256       0.077       0.000      -0.402      -0.101      *

 INTACT     ON
    INTACT&1           0.433       0.083       0.000       0.265       0.591      *
    INTPLAN&1          0.182       0.095       0.025      -0.001       0.373
    AFTERNOON          0.017       0.092       0.427      -0.164       0.201
    EVENING           -0.193       0.084       0.014      -0.348      -0.021      *
    TEMP               0.020       0.075       0.398      -0.118       0.176

 INTPLAN  WITH
    INTACT             0.530       0.071       0.000       0.377       0.654      *

 AFTERNOO WITH
    TEMP               0.438       0.077       0.000       0.273       0.576      *

 EVENING  WITH
    TEMP              -0.245       0.088       0.002      -0.407      -0.065      *
    AFTERNOON         -0.478       0.072       0.000      -0.608      -0.327      *

 Means
    TEMP               0.001       0.091       0.497      -0.184       0.175
    AFTERNOON          0.709       0.104       0.000       0.503       0.906      *
    EVENING            0.634       0.101       0.000       0.442       0.834      *

 Intercepts
    INTPLAN            0.848       0.209       0.000       0.455       1.277      *
    INTACT             0.759       0.233       0.000       0.332       1.244      *

 Variances
    TEMP               1.000       0.000       0.000       1.000       1.000
    AFTERNOON          1.000       0.000       0.000       1.000       1.000
    EVENING            1.000       0.000       0.000       1.000       1.000

 Residual Variances
    INTPLAN            0.524       0.073       0.000       0.379       0.667      *
    INTACT             0.611       0.083       0.000       0.437       0.766      *


    MCMC EFFECTIVE SAMPLE SIZE (ESS) IN ASCENDING ORDER
        LOWEST 10 PARAMETERS
        PARAMETER    ESS
             8      5597
```

```
 1      6416
 2      6565
12      7657
 4     10166
 5     10309
 6     10360
14     10364
16     10364
15     10465
```

4. Mplus Code for the Final Single-Level Models

```
TITLE: 1-level DSEM ID=1
DATA: FILE = "Datei_ordinal.dat";
VARIABLE:
NAMES = VPNr Age Gender School Urban MigBack Edu1 Edu2 Employ1 Employ2 Day Daytime
     IntPlan IntAct JoyMov Perc Temp Accomp Decision Effort hour;
  MISSING=.;
  LAGGED = IntPlan(1) IntAct(1);
  TINTERVAL = hour (1);
  USEVARIABLES=   IntPlan IntAct Temp Perc   afternoon evening   ;
  USEOBSERVATIONS = VPNr EQ 1;! Replace for each ID (1-15)

! Dummy-coding of daytime
DEFINE:
  afternoon = 0;
  IF (Daytime == 2) then afternoon = 1;
  evening = 0;
  IF (Daytime == 3) then evening = 1;

! Log-transformation of DV's
  IntPlan = log(IntPlan);
  IntAct = log(IntAct);

 STANDARDIZE  Temp Perc;

 ANALYSIS:   ESTIMATOR= BAYES;

                FBITERATIONS=(15000);
                PROC= 2;
                BSEED= 46956 ;



MODEL:

 ! gives the x variables variance → becomes a y-variable, missing allowed
  TEMP; PERC; afternoon; evening;


  ! AR(1) for habitual intensity
  IntPlan ON IntPlan&1 (a);

  ! AR(1) for actual intensity
  IntAct  ON IntAct&1 (b);

  ! Cross-lag: habitual at t-1 -> actual at t
  IntAct  ON IntPlan&1 (c);

  ! Concurrent association at time t
  IntPlan WITH IntAct;


  ! Daytime dummies (afternoon, evening vs morning)

  IntPlan IntAct ON afternoon evening;


! Time-varying covariates at time t
  IntAct ON Temp Perc;
```

```
MODEL PRIORS:
  ! Weakly-informative priors for the fixed lag coefficients
  a ~ N(0, 0.25);
  b ~ N(0, 0.25);
  c  ~ N(0, 0.25);

OUTPUT:
  STANDARDIZED;
  TECH8 TECH9;      ! ESS and Rhat


PLOT:
  TYPE = PLOT2;
```

5. Mutli-Level Approach (exploratory, dropped because of unstable between-level variances).

5.1. Caveat on Between-Level Estimates

Although we initially explored between-level effects (age, gender, and temperature – precipitation was not feasible) in the multilevel specification, these parameters exhibited clear signs of weak identification. Posterior standard deviations were large relative to the point estimates, credible intervals were extremely wide (often spanning zero and, for autoregressive slopes, extending beyond the stationarity region), and several variance components were near the boundary with lower limits close to zero—patterns expected under small between-person $N$and Bayesian priors restricted to positive support. Occasional sign changes across time segments further underscore instability. Taken together, these features indicate that the between-level results are largely driven by priors rather than data and cannot be interpreted with confidence. For transparency, summaries are provided in the Supplement, but the main text focuses exclusively on within-person dynamics.

5.2. Results from the Multilevel rDSEM

In contrast to the idiographic single-level approach, which assumes stationarity and estimates dynamics around observed scores (with intercepts capturing person-specific means), the multilevel framework partitions within-person and between-person variation using latent person-mean centering. This centering avoids biases in autoregressive and covariate effects and ensures that within-person parameters reflect deviations from an individual's latent mean rather than the grand mean. Because multilevel models are more vulnerable to systematic time trends, detrending is required before estimating dynamic parameters. To address this, we employed rDSEM, which models lagged relations on residuals rather than raw scores, thereby separating trends from short-term fluctuations and providing unbiased estimates of within-person dynamics.

With the rDSEM, we analyzed the individual trajectories and their fluctuations in the three time periods separately to examine the dynamics of the PA levels.

On a within level, in all three time periods (morning, afternoon and evening), the autoregression between the habitual intensity at timepoint t and t-1 was significant and positive, indicating a significant association between habitual PA at t and t-1 (morning: φ_i^1= 0.332, CRI95% [0.263, 0.397]). Thus, the model predicted that habitual PA at timepoint t was 5.98% higher than one hour before (e.g., morning: increase of 1 standard deviation (SD) of INTHAB (0.903) predicted an average increase of 0.332 SD of INTHAB (→ 0.299 points on the scale of 1-5 (5 points) = (0.299/5)x100=5.98% at time t (one hour later)). Same can be seen in the afternoon (φ_i^1= 0.265, CRI95% [0.177, 0.364]); 0.239 points = 4.8%) and in the evening (φ_i^1=0.4, CRI95% [0.304, 0.488]); 0.444 points = 8.9%) with higher PA levels one hour later, indicating rather stable dynamics of habitual PA over time.

Further, the autoregressive relationships of actual PA at timepoint t and t-1 (φ_i^2) was also positively significant in all three time periods. For instance, in the morning, an increase of 1 SD of INTACT (0.732) resulted in an average increase of 0.238 SD of INTACT (0.174 points = 3.5%). In the afternoon the autoregression was 0.219 points, and in the evening 0.300 points, indicating a higher PA at t compared to t-1 4.3% (afternoon) and 6.0% (evening).

In addition, we analyzed the association of the weather in the region the adolescent is living in and their actual PA behavior. Only in the evening we saw a small, but significant association between the temperature and the actual PA (β_i^1= 0.1; CRI95% [0.013, 0.187]). In more detail, an increase of 4.04°C (SD of TEMP) in the evening compared to the morning temperature was followed by a 1.46% increase of PA levels (0.1 x 0.732 = 0.073 points (1.46%)). Precipitation did not show any significant effects and was excluded from the model in the final analysis.

Furthermore, the association between habitual and actual PA intensity levels was highly significant for all time periods (morning: ψ= 0.615, CRI95% [0.562, 0.663]); afternoon: ψ = 0.5, CRI95% [0.434, 0.559]; evening: ψ = 0.627, CRI95% [0.561, 0.683]).

On the within level, our analysis indicated rather high variance parameters of habitual PA in all time periods (e.g., morning θ= 0.821, CRI95% [0.77, 0.868]) and the residual variance of actual PA levels in each time period (e.g., morning ε= 0.84, CRI95% [0.787, 0.885]).

On the between level, we did not find any significant effects for age, neither for the autoregression slope of habitual (e.g., morning γ= -0.02, CRI95% [-0.354, 0.217]) nor actual PA behavior (e.g., morning γ = 0.041, CRI95% [-0.187, 0.272]). Same can be seen for gender,

with no significant effects on the slopes of the autoregression terms (e.g., INTHAB morning γ = 0.001, CRI95% [-0.468, 0.47]).

Further the association between temperature and actual PA behavior did not reach significant differences for age (e.g., morning γ = 0.009, CRI95% [-0.074, 0.091]) nor gender (e.g., morning γ = -0.001, CRI95% [-0.167, 0.162]).

No significant effects were identified in the habitual activities for age (e.g., morning γ = -0.053, CRI95% [-0.176, 0.063]) or gender (e.g., morning γ =-0.09, CRI95% [-0.347, 0.154]). Only the association between actual PA behavior and gender was significant in the morning (γ = -0.159, CRI95% [-0.347, 0.028]), indicating higher PA levels in the morning in boys than in girls. For age there was no significant effect on actual PA (e.g., morning γ = -0.014, CRI95% [-0.106, 0.081]).

The association between habitual and actual behavior was not significantly different between individuals in none of the different time periods..

Lastly, regarding the residual variances for INTHAB, INTACT, PHI1, PHI2 and BETA1, we see high and significant variance parameter estimates in all time periods.

Dynamics of PA levels: Individual trajectories and their fluctuations in the three time periods (morning, afternoon, evening, presented in each cell).

		Posterior	One-Tailed	CRI 95%		
	Estimate	S.D.	P-Value	Lower 2.5%	Upper 2.5%	Significance
Within-Level *Standardized Estimates Averaged Over Clusters*						
PHI1 φ_i^1 | INTHAB^ ON INTHAB^1	0.332<br>0.265<br>0.400	0.034<br>0.044<br>0.046	0.000<br>0.000	0.263<br>0.177<br>0.304	0.397<br>0.364<br>0.488	***<br>***<br>***
PHI2 φ_i^2 | INTACT^ ON INTACT^1	0.238<br>0.299<br>0.410	0.041<br>0.043<br>0.049	0.000<br>0.000	0.159<br>0.21<br>0.318	0.318<br>0.378<br>0.504	***<br>***<br>***
BETA1 β_i^1 | INTACT ON TEMP	0.029<br>0.036<br>0.100	0.036<br>0.043<br>0.044	0.203<br>0.198<br>0.013	-0.04<br>-0.046<br>0.013	0.1<br>0.12<br>0.187	**
ψ | INTHAB WITH INTACT	0.615<br>0.5<br>0.627	0.025<br>0.032<br>0.031	0.000<br>0.000	0.562<br>0.434<br>0.561	0.663<br>0.559<br>0.683	***<br>***<br>***
Variances						
θ | INTHAB	0.821<br>0.872<br>0.802	0.025<br>0.024<br>0.035	0.000<br>0.000<br>0.000	0.77<br>0.82<br>0.731	0.868<br>0.915<br>0.868	***<br>***<br>***
Residual Variances						
INTACT	0.84	0.025	0.000	0.787	0.885	***

	0.818<br>0.802	0.029<br>0.035	0.000<br>0.000	0.752<br>0.731	0.869<br>0.868	***<br>***
TEMP	1.000<br>1.000<br>1.000	0.000<br>0.000<br>0.000	0.000<br>0.000<br>0.000	1.000<br>1.000<br>1.000	1.000<br>1.000<br>1.000	
Between Level Unstandardized means and variances						
γ | *PHI1 ON*						
AGE	-0.002<br>0.057<br>0.041	0.119<br>0.117<br>0.112	0.43<br>0.306<br>0.354	-0.254<br>-0.173<br>-0.18	0.217<br>0.29<br>0.262	
GENDER	0.001<br>-0.047<br>0.06	0.237<br>0.236<br>0.225	0.499<br>0.422<br>0.393	-0.468<br>-0.514<br>-0.383	0.47<br>0.418<br>0.507	
γ | *PHI2 ON*						
AGE	0.041<br>0.055<br>-0.002	0.116<br>0.114<br>0.119	0.355<br>0.307<br>0.433	-0.187<br>-0.168<br>-0.258	0.272<br>0.283<br>0.212	
GENDER	-0.124<br>-0.141<br>-0.078	0.235<br>0.232<br>0.239	0.295<br>0.267<br>0.369	-0.586<br>-0.597<br>-0.547	0.339<br>0.314<br>0.398	
γ | *BETA1 ON*						
AGE	0.009<br>-0.004<br>-0.036	0.041<br>0.043<br>0.173	0.414<br>0.461<br>0.417	-0.074<br>-0.09<br>-0.379	0.091<br>0.081<br>0.308	
GENDER	-0.001<br>0.002<br>0.01	0.083<br>0.086<br>0.346	0.494<br>0.498<br>0.488	-0.167<br>-0.168<br>-0.68	0.162<br>0.171<br>0.693	
γ | *INTHAB ON*						
AGE	-0.053<br>0.07<br>0.073	0.06<br>0.118<br>0.106	0.167<br>0.258<br>0.224	-0.176<br>-0.165<br>-0.134	0.063<br>0.307<br>0.289	
GENDER	-0.09<br>-0.189<br>-0.106	0.126<br>0.236<br>0.213	0.218<br>0.186<br>0.292	-0.347<br>-0.668<br>-0.529	0.154<br>0.27<br>0.319	
γ | *INTACT ON*						
AGE	-0.014<br>0.041<br>0.052	0.048<br>0.066<br>0.111	0.379<br>0.253<br>0.304	-0.106<br>-0.008<br>-0.163	0.081<br>0.175<br>0.279	
GENDER	-0.159<br>-0.051<br>-0.067	0.095<br>0.13<br>0.217	0.044<br>0.334<br>0.365	-0.347<br>-0.314<br>-0.505	0.028<br>0.204<br>0.364	*
INTACT<br>WITH<br>INTHAB	0.004<br>0.028<br>0.064	0.019<br>0.061<br>0.098	0.335<br>0.169<br>0.062	-0.017<br>-0.035<br>-0.018	0.052<br>0.183<br>0.324	
Means						
TEMP	-0.011<br>-0.007<br>0.024	0.685<br>0.881<br>0.738	0.493<br>0.497<br>0.486	-1.369<br>-1.617<br>-1.444	1.358<br>1.611<br>1.47	

Intercepts						
	2.328	0.196	0	1.946	2.73	***
INTHAB	2.956	0.366	0	2.243	3.71	***
	2.072	0.327	0	1.43	2.729	***
	2.440	0.151	0	2.143	2.735	***
INTACT	2.483	0.206	0	2.085	2.898	***
	2.092	0.34	0	1.42	2.78	***
	0.331	0.365	0.178	-0.392	1.047	
PHI1	0.329	0.364	0.179	-0.385	1.042	
	0.315	0.349	0.18	-0.377	0.999	
	0.42	0.362	0.118	-0.295	1.130	
PHI2	0.505	0.365	0.078	-0.204	1.209	
	0.525	0.367	0.075	-0.213	1.240	
	0.011	0.129	0.465	-0.245	0.266	
BETA1	0.008	0.134	0.476	-0.254	0.272	
	0.008	0.537	0.493	-1.046	1.078	
Variances						
	6.007	3.477	0.000	2.813	15.802	***
TEMP	8.559	4.887	0.000	4.076	22.389	***
	7.036	4.188	0.000	3.266	18.568	***
Residual Variances						
	0.022	0.038	0.000	0.002	0.128	***
INTHAB	0.141	0.156	0.000	0.043	0.537	***
	0.089	0.112	0.000	0.16	0.401	***
	0.011	0.019	0.000	0.001	0.062	***
INTACT	0.025	0.04	0.000	0.003	0.134	***
	0.11	0.122	0.000	0.03	0.442	***
	0.175	0.068	0.000	0.098	0.358	***
PHI1	0.162	0.063	0.000	0.09	0.331	***
	0.146	0.057	0.000	0.082	0.299	***
	0.166	0.064	0.000	0.092	0.339	***
PHI2	0.161	0.063	0.000	0.089	0.33	***
	0.164	0.065	0.000	0.091	0.339	***
	0.023	0.009	0.000	0.013	0.046	***
BETA1	0.023	0.009	0.000	0.013	0.047	***
	0.418	0.157	0.000	0.235	0.836	***

*Note: INTHAB = habitual intensity; INTACT = actual Intensity; TEMP = Temperature. *** = significant in the 99% CRI, ** = significant in the 95% CRI, * = significant in the 90% CRI.
